# Supplementary material for: Oxalactam A, a Novel Macrolactam with Potent Anti-Rhizoctonia solani Activity from the Endophytic Fungus Penicillium oxalicum
Source: Molecules. 2022 Dec 12;27(24):8811. doi: 10.3390/molecules27248811 (PMC9788486; doi:10.3390/molecules27248811)
Supplement: Supplementary file 1 [file molecules-27-08811-s001.zip › molecules-1966471-supplementary.pdf]

Supporting information

Oxalactam A, a novel macrolactam with potent anti-*Rhizoctonia solani* activity from the endophytic fungus *Penicillium oxalicum*

Ruizhen Zhang [1,†], Yingrun Ma [1,†], Ming-Ming Xu [1], Xinyi Wei [1], Cheng-Bin Yang [1], Fei Zeng [1], Jin-Ao Duan [1], Chun-Tao Che [2,*], Junfei Zhou [1,*] and Ming Zhao [1,2,*]

1 National and Local Collaborative Engineering Center of Chinese Medicinal Resources Industrialization and Formulae Innovative Medicine, Jiangsu Collaborative Innovation Center of Chinese Medicinal Resources Industrialization, School of Pharmacy, Nanjing University of Chinese Medicine, Nanjing, 210023, China

2 Department of Pharmaceutical Sciences, College of Pharmacy, The University of Illinois at Chicago, Chicago, IL 60612, USA

* Correspondence: chect@uic.edu (C.-T.C.); 300524@njucm.edu.cn (J.Z.); mingzhao@njucm.edu.cn (M.Z.); Tel.: +86-15895865208 (M.Z.)

† These authors contributed equally to this work.

Contents

Fungus identification 1
Figure S1. Flow chart of extraction and isolation. 3
Figure S2. *Penicillium oxalicum* resisted the growth of four plant pathogenic fungi strains (Left of A−D: blank group; right of A−D: *Penicillium oxalicum*-treated group). 4
Table S1. *Penicillium oxalicum* resisted the growth of four plant pathogenic fungi strains. 4
Table S2. Cartesian coordinate for the lowest energy conformer of **1** calculated at TD/B3LYP/6-311G(d,p) level of theory in the gas phase. 5
Table S3. Experimental and calculated chemical shifts of 1 for DP4+ probability statistical analysis. 7
Figure S3. The result of ANNs analysis for (3*R**,15*S**,16*S**)-**1**, and corresponding 18 parameters ... 8
Figure S4. The result of ANNs analysis for (3*R**,15*R**,16*S**)-**1**, and corresponding 18 parameters ... 8
Figure S5. The result of ANNs analysis for (3*S**,15*S**,16*S**)-**1**, and corresponding 18 parameters 9
Figure S6. The result of ANNs analysis for (3*S**,15*R**,16*S**)-**1**, and corresponding 18 parameters ... 9
Figure S7. ^{1}H NMR (500 MHz, methanol-d_4) of compound **1** 10
Figure S8. ^{13}C NMR (125 MHz, methanol-d_4) of compound **1** 10
Figure S9. DEPT135 (125 MHz, methanol-d_4) of compound **1** 11
Figure S10. ^{1}H−^{1}H COSY (500 MHz, methanol-d_4) of compound **1** 11
Figure S11. HSQC (methanol-d_4) of compound **1** (^{1}H: 500 MHz, ^{13}C: 125 MHz) 12
Figure S12. HMBC (methanol-d_4) of compound **1** (^{1}H: 500 MHz, ^{13}C: 125 MHz) 12
Figure S13. NOESY (500 MHz, methanol-d_4) of compound **1** 12
Figure S14. (+)-HR-ESI-MS of compound **1** 14
Figure S15. UV of compound **1** 14
Figure S16. ^{1}H NMR (500 MHz, methanol-d_4) of compound **2** 14
Figure S17. ^{13}C NMR (125 MHz, methanol-d_4) of compound **2** 15
Figure S18. ^{1}H NMR (500 MHz, methanol-d_4) of compound **3** 16
Figure S19. ^{13}C NMR (125 MHz, methanol-d_4) of compound **3** 16
Figure S20. ^{1}H NMR (500 MHz, methanol-d_4) of compound **4** 17
Figure S21. ^{13}C NMR (125 MHz, methanol-d_4) of compound **4** 17
Figure S22. ^{1}H NMR (500 MHz, methanol-d_4) of compound **5** 18
Figure S23. ^{13}C NMR (125 MHz, methanol-d_4) of compound **5** 18
Figure S24. ^{1}H NMR (500 MHz, methanol-d_4) of compound **6** 19
Figure S25. ^{13}C NMR (125 MHz, methanol-d_4) of compound **6** 19
Figure S26. ^{1}H NMR (500 MHz, pyridine-d_5) of compound **7** 20
Figure S27. ^{13}C NMR (125 MHz, pyridine-d_5) of compound **7**. 20
Figure S28. HPLC chromatogram of the derivative of the standard *L*-Glucose. 21
Figure S29. HPLC chromatogram of the derivative of the standard *D*-Glucose. 21
Figure S30. HPLC chromatogram of derivative of the hydrolysate of compound **1** 22
Figure S31. Compounds 1−7 and carbendazim (100 μM) inhibit the growth of *Rhizoctonia solani*. 22
Figure S32. Growth inhibition of *Rhizoctonia solani* with different concentrations of compound **1**. 22

Fungus identification

The test endophytic fungus was identified as *Penicillium oxalicum* based on its morphological characteristics and 16S rRNA gene sequence by Shanghai Shenggong Bioengineering Co., Ltd., the 16S rRNA gene sequence is TTGATATGCTTAAGTTCAGCGGGTATCCCTACCTGATCCGAG GTCAACCTGGTTAAGATTGATGGTGTTCGCCGGCGGGCGCCGGCCGGGCCTACAGAGCG GGTGACGAAGCCCCATACGCTCGAGGACCGGACGCGGTGCCGCCGCTGCCTTTCGGGCC CGCCCCCCGGAAGCGGGGGGCGAGAGCCCAACACACAAGCCGTGCTTGAGGGCAGCAA TGACGCTCGGACAGGCATGCCCCCCGGAATACCAGGGGGCGCAATGTGCGTTCAAAGAC TCGATGATTCACTGAATTCTGCAATTCACATTACTTATCGCATTTCGCTGCGTTCTTCATCG ATGCCGGAACCAAGAGATCCGTTGTTGAAAGTTTTAACTGATTTAGTCAAGTACTCAGAC TGCAATCTTCAGACAAGAGTTCGTTTGTGTGTCTTCGGCGGGCGCGGGCCCGGGGGCGG ATGCCCCCCGGCGGCCGTGAGGCGGGCCCGCCGAAGCAACAAGGTACGATAAACACGG GTGGGAGGTTGGACCCAGAGGGCCCTCACTCGGTAATGATCCTTCCGCAG (566 bp).

The identified result is as follows:

Description	Max Score	Total Score	Query Cover	E value	Per. Ident	Accession
Penicillium oxalicum strain 6-1F small subunit ribosomal RNA gene, partial sequence; internal transcribed spacer 1, 5.8S ribosomal RNA gene, and internal transcribed spacer 2, complete sequence; and large subunit ribosomal RNA gene, partial sequence	1046	1046	100%	0.0	100.00%	MW077100.1
Aspergillus aculeatus strain 3-18F small subunit ribosomal RNA gene, partial sequence; internal transcribed spacer 1, 5.8S ribosomal RNA gene, and internal transcribed spacer 2, complete sequence; and large subunit ribosomal RNA gene, partial sequence	1046	1046	100%	0.0	100.00%	MW077088.1
Penicillium oxalicum strain 3-16F small subunit ribosomal RNA gene, partial sequence; internal transcribed spacer 1, 5.8S ribosomal RNA gene, and internal transcribed spacer 2, complete sequence; and large subunit ribosomal RNA gene, partial sequence	1046	1046	100%	0.0	100.00%	MW077086.1
Nigrospora sphaerica strain 5-1F small subunit ribosomal RNA gene, partial sequence; internal transcribed spacer 1, 5.8S ribosomal RNA gene, and internal transcribed spacer 2, complete sequence; and large subunit ribosomal RNA gene, partial sequence	1046	1046	100%	0.0	100.00%	MW077068.1
Penicillium oxalicum strain 2-5F small subunit ribosomal	1046	1046	100%	0.0	100.00%	MW077050.1

Description	Max Score	Total Score	Query Cover	E value	Per. Ident	Accession
RNA gene, partial sequence; internal transcribed spacer 1, 5.8S ribosomal RNA gene, and internal transcribed spacer 2, complete sequence; and large subunit ribosomal RNA gene, partial sequence						
Penicillium oxalicum strain 2-4F small subunit ribosomal RNA gene, partial sequence; internal transcribed spacer 1, 5.8S ribosomal RNA gene, and internal transcribed spacer 2, complete sequence; and large subunit ribosomal RNA gene, partial sequence	1046	1046	100%	0.0	100.00%	MW077049.1
Penicillium oxalicum strain 2-3F small subunit ribosomal RNA gene, partial sequence; internal transcribed spacer 1, 5.8S ribosomal RNA gene, and internal transcribed spacer 2, complete sequence; and large subunit ribosomal RNA gene, partial sequence	1046	1046	100%	0.0	100.00%	MW077048.1
Penicillium oxalicum strain 2-2F small subunit ribosomal RNA gene, partial sequence; internal transcribed spacer 1, 5.8S ribosomal RNA gene, and internal transcribed spacer 2, complete sequence; and large subunit ribosomal RNA gene, partial sequence	1046	1046	100%	0.0	100.00%	MW077047.1
Penicillium sp. isolate SZMC27118 small subunit ribosomal RNA gene, partial sequence; internal transcribed spacer 1, 5.8S ribosomal RNA gene, and internal transcribed spacer 2, complete sequence; and large subunit ribosomal RNA gene, partial sequence	1046	1046	100%	0.0	100.00%	MT997202.1
Penicillium oxalicum isolate Z1 small subunit ribosomal RNA gene, partial sequence; internal transcribed spacer 1, 5.8S ribosomal RNA gene, and internal transcribed spacer 2, complete sequence; and large subunit ribosomal RNA gene, partial sequence	1046	1046	100%	0.0	100.00%	MT795727.1

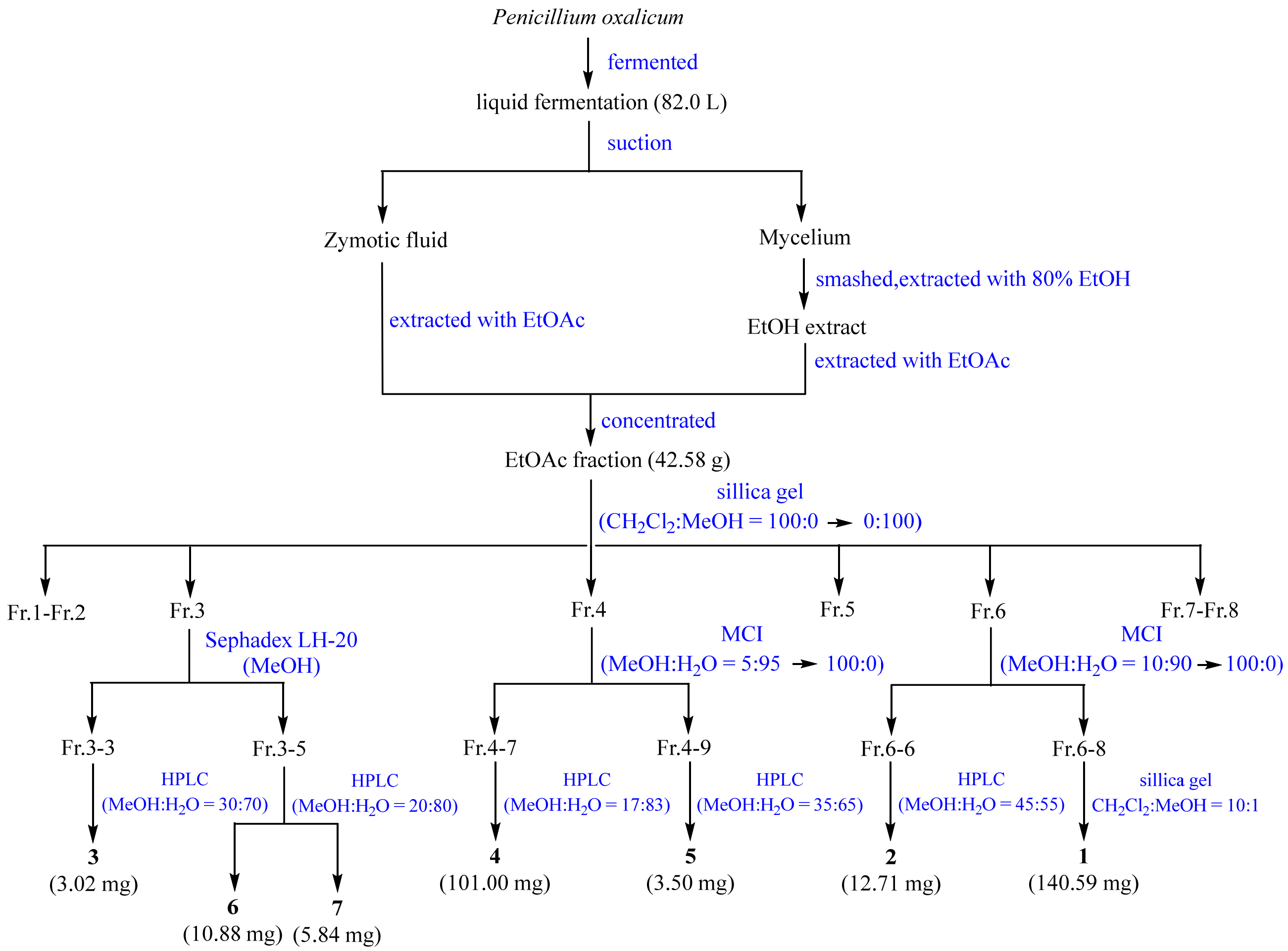

Figure S1. Flow chart of extraction and isolation.

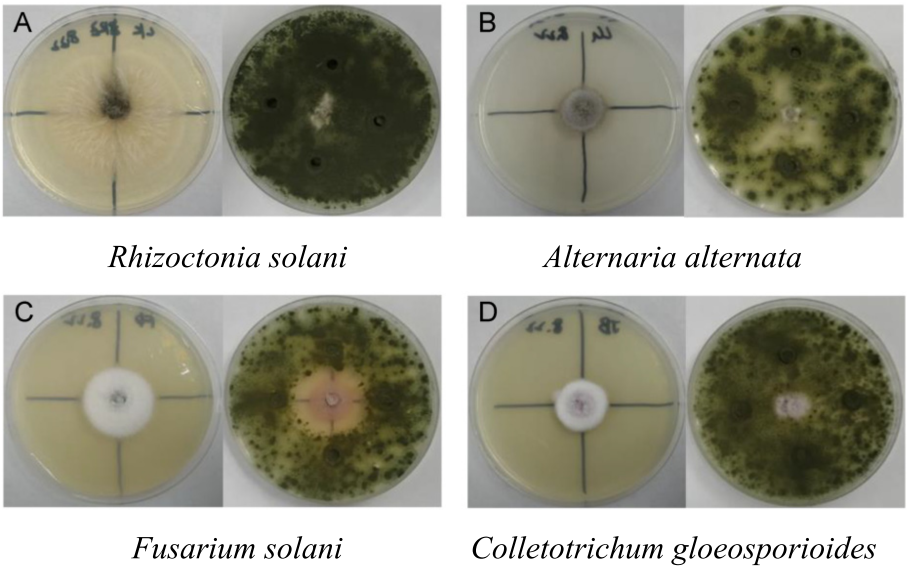

Figure S2. *Penicillium oxalicum* resisted the growth of four plant pathogenic fungi strains (Left of A−D: blank group; right of A−D: *Penicillium oxalicum*-treated group).

Table S1. *Penicillium oxalicum* resisted the growth of four plant pathogenic fungi strains.

Strain	Inhibition rate (%)
Rhizoctonia solani	96.83 ± 1.19
Alternaria alternata	83.33 ± 1.39
Fusarium solani	50.67 ± 4.99
Colletotrichum gloeosporioides	76.04 ± 1.47

Table S2. Cartesian coordinate for the lowest energy conformer of **1** calculated at TD/B3LYP/6-311G(d,p) level of theory in the gas phase.

Center number	Atomic number	Atomic type	Coordinates (Angstroms)		
			X	Y	Z
1	6	0	-0.9139	-2.73884	1.114038
2	6	0	-0.77555	-3.23078	-0.3526
3	6	0	-2.11285	-3.11669	-1.04262
4	6	0	-2.31535	-2.4007	-2.15141
5	7	0	-0.8874	-1.37729	1.215678
6	6	0	-3.65999	-2.12229	-2.77092
7	6	0	-4.21833	-0.74158	-2.34803
8	6	0	-3.31855	0.453999	-2.73833
9	6	0	-3.69691	1.739884	-2.02721
10	6	0	-2.93638	2.181279	-1.01094
11	6	0	-3.18887	3.343385	-0.08781
12	6	0	-3.77944	2.894613	1.28091
13	6	0	-2.84101	2.09065	2.149921
14	6	0	-2.75052	0.75579	2.146786
15	6	0	-1.89467	-0.06965	3.083638
16	6	0	-0.60933	-0.67227	2.457201
17	6	0	-4.96241	2.408002	-2.50874
18	8	0	-1.45992	0.659509	4.230495
19	6	0	0.494268	0.362572	2.222807
20	8	0	1.707668	-0.33157	1.89877
21	8	0	-1.04788	-3.50217	2.059975
22	8	0	0.181257	-2.46234	-1.06504
23	6	0	2.275283	-0.05938	0.673944
24	6	0	3.321475	-1.1331	0.364571
25	6	0	4.059023	-0.76686	-0.91759
26	6	0	4.665464	0.629018	-0.84227
27	6	0	3.561163	1.632114	-0.45616
28	8	0	2.924099	1.216681	0.749118
29	6	0	4.100948	3.029156	-0.15935
30	8	0	4.808709	3.4854	-1.31768
31	8	0	5.231616	0.885829	-2.11783
32	8	0	5.041047	-1.77349	-1.13176
33	8	0	2.692882	-2.40729	0.214725
34	1	0	-0.47408	-4.28637	-0.27297
35	1	0	-2.93681	-3.62479	-0.54097
36	1	0	-1.45001	-1.92763	-2.60885
37	1	0	-0.69813	-0.89634	0.344619
38	1	0	-4.38136	-2.89318	-2.47087
39	1	0	-3.58606	-2.16537	-3.86702
40	1	0	-5.22054	-0.61337	-2.7782

41	1	0	-4.33944	-0.74486	-1.25705
42	1	0	-2.27954	0.21174	-2.49121
43	1	0	-3.35969	0.59176	-3.82968
44	1	0	-2.05161	1.592933	-0.76584
45	1	0	-3.88452	4.061655	-0.53547
46	1	0	-2.25075	3.888168	0.088837
47	1	0	-4.09289	3.791592	1.831846
48	1	0	-4.68659	2.312254	1.079748
49	1	0	-2.20146	2.652382	2.832022
50	1	0	-3.37169	0.177624	1.463398
51	1	0	-2.49892	-0.93793	3.393617
52	1	0	-0.23054	-1.4136	3.167767
53	1	0	-5.17942	3.344843	-1.9897
54	1	0	-4.89718	2.626188	-3.58372
55	1	0	-5.83163	1.749251	-2.37924
56	1	0	-2.25365	1.01677	4.658835
57	1	0	0.222016	1.058687	1.41852
58	1	0	0.662766	0.943832	3.130693
59	1	0	1.050535	-2.55034	-0.61807
60	1	0	1.519179	-0.02418	-0.13162
61	1	0	4.025355	-1.1642	1.206856
62	1	0	3.333585	-0.76918	-1.74859
63	1	0	5.437531	0.639082	-0.05428
64	1	0	2.837095	1.688527	-1.28614
65	1	0	4.763298	2.969776	0.715877
66	1	0	3.26582	3.69892	0.085292
67	1	0	5.246876	4.321451	-1.10086
68	1	0	5.453743	1.835423	-2.13529
69	1	0	5.528399	-1.51461	-1.93159
70	1	0	3.364891	-2.98041	-0.19571

Table S3. Experimental and calculated chemical shifts of **1** for DP4+ probability statistical analysis.

Functional	Solvent	Basis Set				Type of Data			
mPW1PW91	Methanol	6-311G(d,p)				Scaled Shifts			
DP4+		3*R**,15*S**,16*S**-**1**		3*S**,15*S**,16*S**-**1**		3*S**,15*R**,16*S**-**1**		3*R**,15*R**,16*S**-**1**	
Bayes's theorem probability		97.08%		0%		2.92%		0%	
Nuclei	$\delta_{experimental}$	$\delta_{calculated}$	$\delta_{corrected}$	$\delta_{calculated}$	$\delta_{corrected}$	$\delta_{calculated}$	$\delta_{corrected}$	$\delta_{calculated}$	$\delta_{corrected}$
C-2	175.6	180.8	173.3	176.1	170.9	180.3	171.8	182.3	174.3
C-3	74.2	77.1	73.3	75.6	72.9	78.7	74.0	76.9	72.2
C-4	129.2	134.8	129.0	133.1	128.9	138.2	131.2	132.9	126.4
C-5	134.8	143.4	137.3	145.4	140.9	145.1	137.9	148.0	141.1
C-6	33.6	37.0	34.6	33.7	32.1	33.5	30.4	35.7	32.3
C-7	29.3	33.7	31.5	33.3	31.7	32.2	29.2	35.2	31.8
C-8	41.0	41.2	38.7	37.1	35.4	38.9	35.6	38.2	34.7
C-9	136.9	148.4	142.1	146.4	142.0	147.5	140.2	149.8	142.9
C-10	125.0	132.5	126.7	133.1	128.9	131.1	124.4	132.8	126.3
C-11	28.8	29.5	27.4	27.0	25.6	31.9	28.9	33.1	29.8
C-12	33.9	39.0	36.5	34.1	32.5	36.6	33.4	37.4	33.9
C-13	134.7	138.4	132.4	138.0	133.7	143.2	136.1	138.9	132.3
C-14	131.2	137.3	131.3	133.8	129.6	138.9	131.9	138.8	132.2
C-15	73.0	75.2	71.5	74.6	72.0	76.4	71.7	76.6	71.9
C-16	54.7	58.1	55.0	57.1	55.0	57.6	53.6	60.8	56.6
C-17	69.8	71.7	68.1	71.5	69.0	74.8	70.2	74.8	70.2
C-18	16.3	16.9	15.2	26.1	24.7	26.8	24.0	27.5	24.3
C-1'	104.9	107.1	102.3	105.0	101.5	108.6	102.7	110.0	104.2
C-2'	75.1	79.5	75.6	79.6	76.8	79.3	74.5	77.5	72.8
C-3'	78.1	80.1	76.2	81.2	78.4	81.9	77.0	79.2	74.4
C-4'	71.7	74.5	70.8	71.7	69.1	76.3	71.6	75.6	71.0
C-5'	78.0	79.9	76.0	80.7	77.9	79.7	74.9	78.9	74.1
C-6'	62.8	71.4	67.8	65.4	63.0	71.7	67.2	67.3	62.9

δ Exp (in descending order)	σ calc (in ascending order)	Type "x" if sp or sp2 hybridized
175.6	28.26	x
136.9	61.37	x
134.8	69.38	x
134.7	70.65	x
131.2	71.37	x
129.2	73.87	x
125	76.49	x
104.9	95.41	
78.1	120.59	
78	122.46	
75.1	123.03	
74.2	123.64	
73	125.96	
71.7	126.12	
69.8	129.1	
62.8	130.38	
54.7	141.39	
41	158.66	
33.9	159.97	
33.6	162.6	
29.3	165.83	
28.8	169.27	
16.3	180.95	

Calculated Chemical Shifts			
Unscaled		Scaled	
MSTD	TMS	MSTD	TMS
174.0	166.5	175.0	176.3
140.9	133.4	142.3	141.2
132.9	125.4	134.4	132.7
131.7	124.1	133.1	131.3
130.9	123.4	132.4	130.6
128.4	120.9	129.9	127.9
125.8	118.3	127.3	125.1
99.4	99.4	101.2	105.0
74.2	74.2	76.3	78.3
72.3	72.3	74.4	76.3
71.7	71.7	73.9	75.7
71.1	71.1	73.3	75.0
68.8	68.8	71.0	72.6
68.6	68.6	70.8	72.4
65.7	65.7	67.9	69.2
64.4	64.4	66.6	67.9
53.4	53.4	55.7	56.2
36.1	36.1	38.6	37.8
34.8	34.8	37.4	36.5
32.2	32.2	34.8	33.7
28.9	28.9	31.6	30.2
25.5	25.5	28.2	26.6
13.8	13.8	16.6	14.2
-	-	-	-
-	-	-	-
-	-	-	-
-	-	-	-
-	-	-	-
-	-	-	-
-	-	-	-

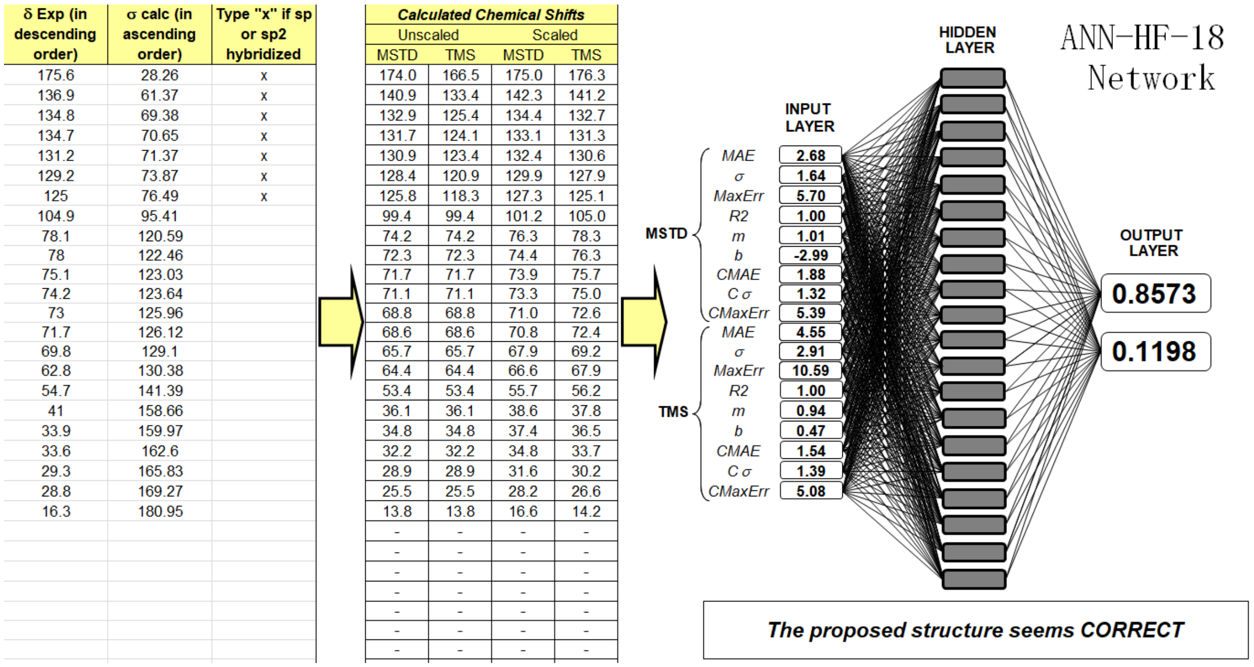

Figure S3. The result of ANNs analysis for (3*R**,15*S**,16*S**)-**1**, and corresponding 18 parameters.

δ Exp (in descending order)	σ calc (in ascending order)	Type "x" if sp or sp2 hybridized
175.6	34.3	x
136.9	64.74	x
134.8	65.14	x
134.7	68.74	x
131.2	70.87	x
129.2	71.5	x
125	74.71	x
104.9	94.21	
78.1	119.87	
78	120.2	
75.1	122.43	
74.2	124.86	
73	125.61	
71.7	129.05	
69.8	130.16	
62.8	131.62	
54.7	142.35	
41	161.93	
33.9	162.49	
33.6	163.55	
29.3	164.76	
28.8	165.03	
16.3	171.9	

Calculated Chemical Shifts			
Unscaled		Scaled	
MSTD	TMS	MSTD	TMS
168.0	160.5	170.2	171.1
137.6	130.0	139.6	138.2
137.2	129.6	139.2	137.8
133.6	126.0	135.6	133.9
131.4	123.9	133.4	131.6
130.8	123.3	132.8	130.9
127.6	120.1	129.6	127.5
100.6	100.6	102.4	106.4
74.9	74.9	76.6	78.7
74.6	74.6	76.3	78.3
72.3	72.3	74.0	75.9
69.9	69.9	71.6	73.3
69.2	69.2	70.9	72.5
65.7	65.7	67.4	68.8
64.6	64.6	66.3	67.6
63.1	63.1	64.8	66.0
52.4	52.4	54.0	54.4
32.8	32.8	34.4	33.2
32.3	32.3	33.8	32.6
31.2	31.2	32.7	31.5
30.0	30.0	31.5	30.2
29.7	29.7	31.3	29.9
22.9	22.9	24.3	22.5
-	-	-	-
-	-	-	-
-	-	-	-
-	-	-	-
-	-	-	-
-	-	-	-
-	-	-	-

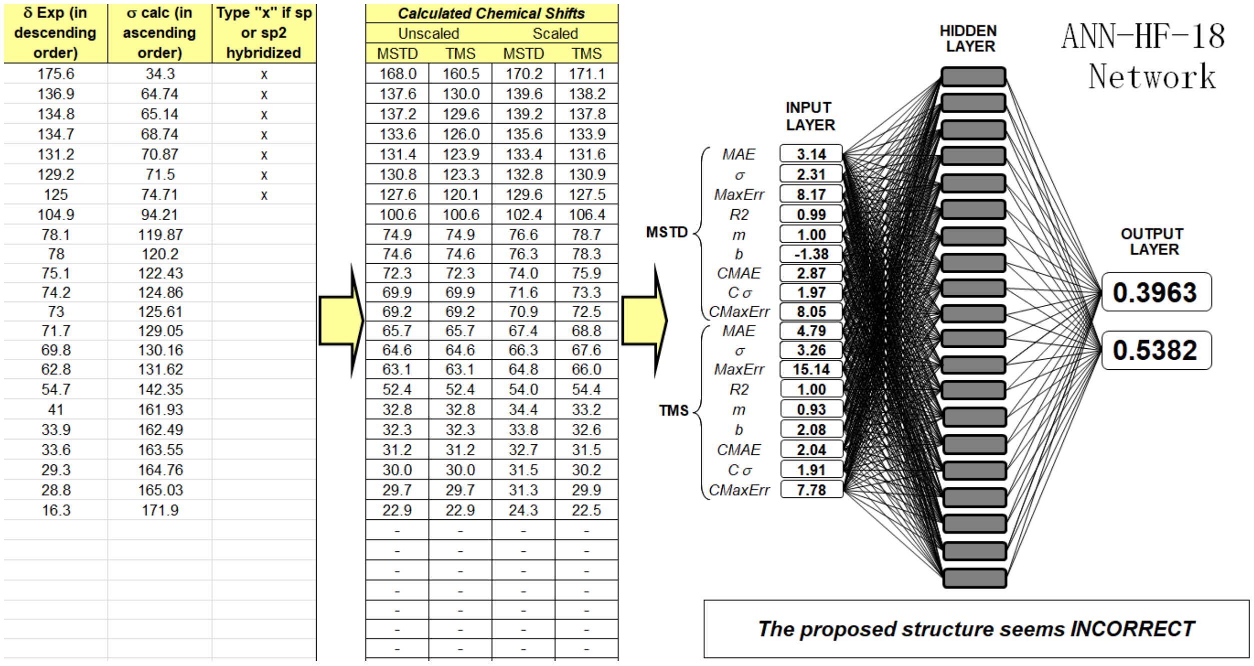

Figure S4. The result of ANNs analysis for (3*R**,15*R**,16*S**)-**1**, and corresponding 18 parameters.

δ Exp (in descending order)	σ calc (in ascending order)	Type "x" if sp or sp2 hybridized	Calculated Chemical Shifts: Unscaled MSTD	Unscaled TMS	Scaled MSTD	Scaled TMS
175.6	28.44	x	173.9	166.3	173.6	174.8
136.9	62.67	x	139.6	132.1	139.8	138.5
134.8	67.89	x	134.4	126.9	134.6	132.9
134.7	68.39	x	133.9	126.4	134.1	132.4
131.2	68.56	x	133.7	126.2	134.0	132.2
129.2	70.66	x	131.6	124.1	131.9	130.0
125	77.01	x	125.3	117.8	125.6	123.3
104.9	92.23		102.5	102.5	103.1	107.1
78.1	118.05		76.7	76.7	77.7	79.7
78	121.48		73.3	73.3	74.3	76.1
75.1	122.89		71.9	71.9	72.9	74.6
74.2	123.07		71.7	71.7	72.7	74.4
73	123.22		71.5	71.5	72.6	74.3
71.7	123.42		71.3	71.3	72.4	74.1
69.8	125.18		69.6	69.6	70.6	72.2
62.8	129.27		65.5	65.5	66.6	67.9
54.7	141.49		53.3	53.3	54.5	54.9
41	161.61		33.2	33.2	34.7	33.6
33.9	163.27		31.5	31.5	33.0	31.8
33.6	166.42		28.3	28.3	29.9	28.5
29.3	166.42		28.3	28.3	29.9	28.5
28.8	166.56		28.2	28.2	29.8	28.3
16.3	171.91		22.9	22.9	24.5	22.6
			-	-	-	-
			-	-	-	-
			-	-	-	-
			-	-	-	-
			-	-	-	-
			-	-	-	-
			-	-	-	-

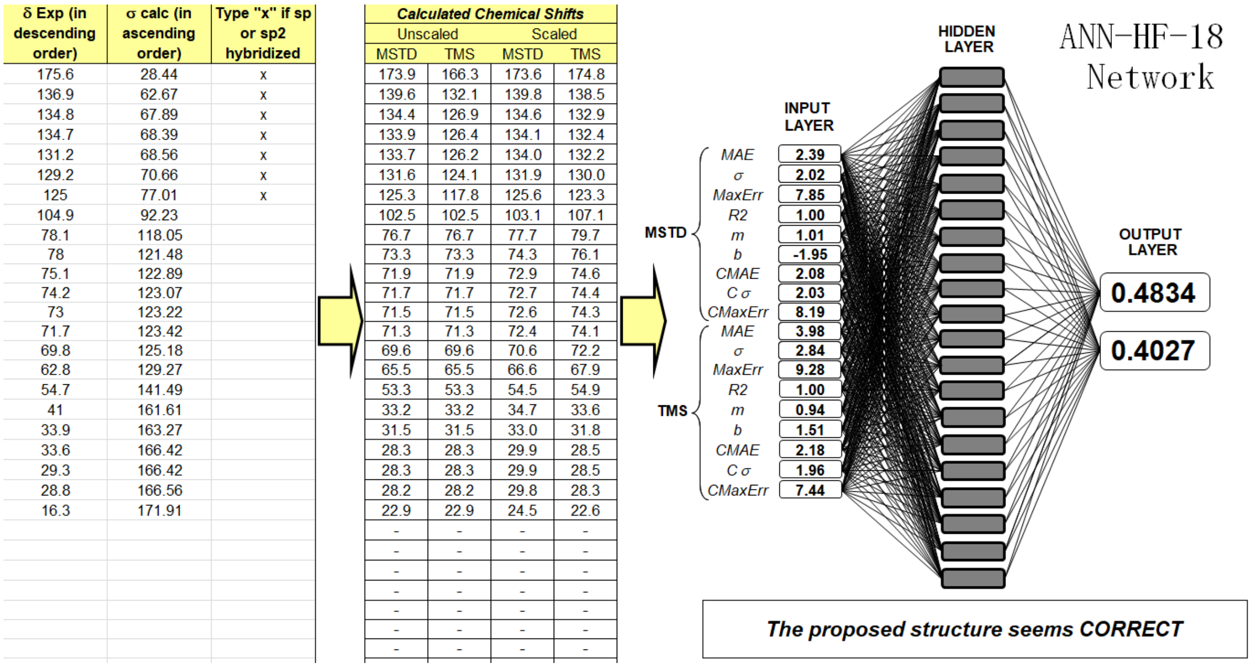

Figure S5. The result of ANNs analysis for (3*S**,15*S**,16*S**)-**1**, and corresponding 18 parameters.

δ Exp (in descending order)	σ calc (in ascending order)	Type "x" if sp or sp2 hybridized	Calculated Chemical Shifts: Unscaled MSTD	Unscaled TMS	Scaled MSTD	Scaled TMS
175.6	27.87	x	174.4	166.9	175.0	176.4
136.9	62.28	x	140.0	132.5	140.8	139.6
134.8	64.54	x	137.8	130.2	138.6	137.2
134.7	70.05	x	132.3	124.7	133.1	131.3
131.2	71.23	x	131.1	123.5	131.9	130.0
129.2	72.09	x	130.2	122.7	131.0	129.1
125	76.27	x	126.0	118.5	126.9	124.6
104.9	95.42		99.3	99.3	100.3	104.1
78.1	120.47		74.3	74.3	75.4	77.3
78	120.75		74.0	74.0	75.1	77.0
75.1	123.31		71.5	71.5	72.6	74.3
74.2	123.31		71.5	71.5	72.6	74.3
73	124.14		70.6	70.6	71.8	73.4
71.7	124.87		69.9	69.9	71.0	72.6
69.8	125.75		69.0	69.0	70.2	71.7
62.8	133.85		60.9	60.9	62.1	63.0
54.7	137.94		56.8	56.8	58.0	58.7
41	161.03		33.7	33.7	35.0	34.0
33.9	163.02		31.7	31.7	33.1	31.8
33.6	163.87		30.9	30.9	32.2	30.9
29.3	163.94		30.8	30.8	32.2	30.9
28.8	166.99		27.8	27.8	29.1	27.6
16.3	171.52		23.2	23.2	24.6	22.8
			-	-	-	-
			-	-	-	-
			-	-	-	-
			-	-	-	-
			-	-	-	-
			-	-	-	-
			-	-	-	-

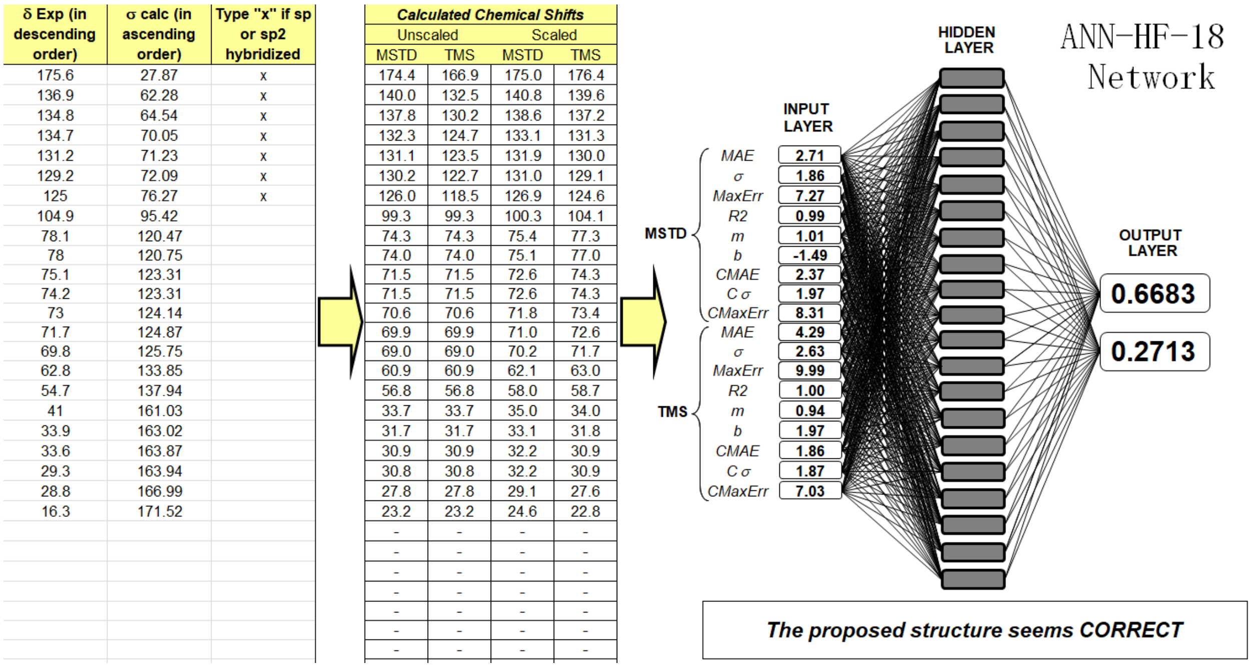

Figure S6. The result of ANNs analysis for (3*S**,15*R**,16*S**)-**1**, and corresponding 18 parameters.

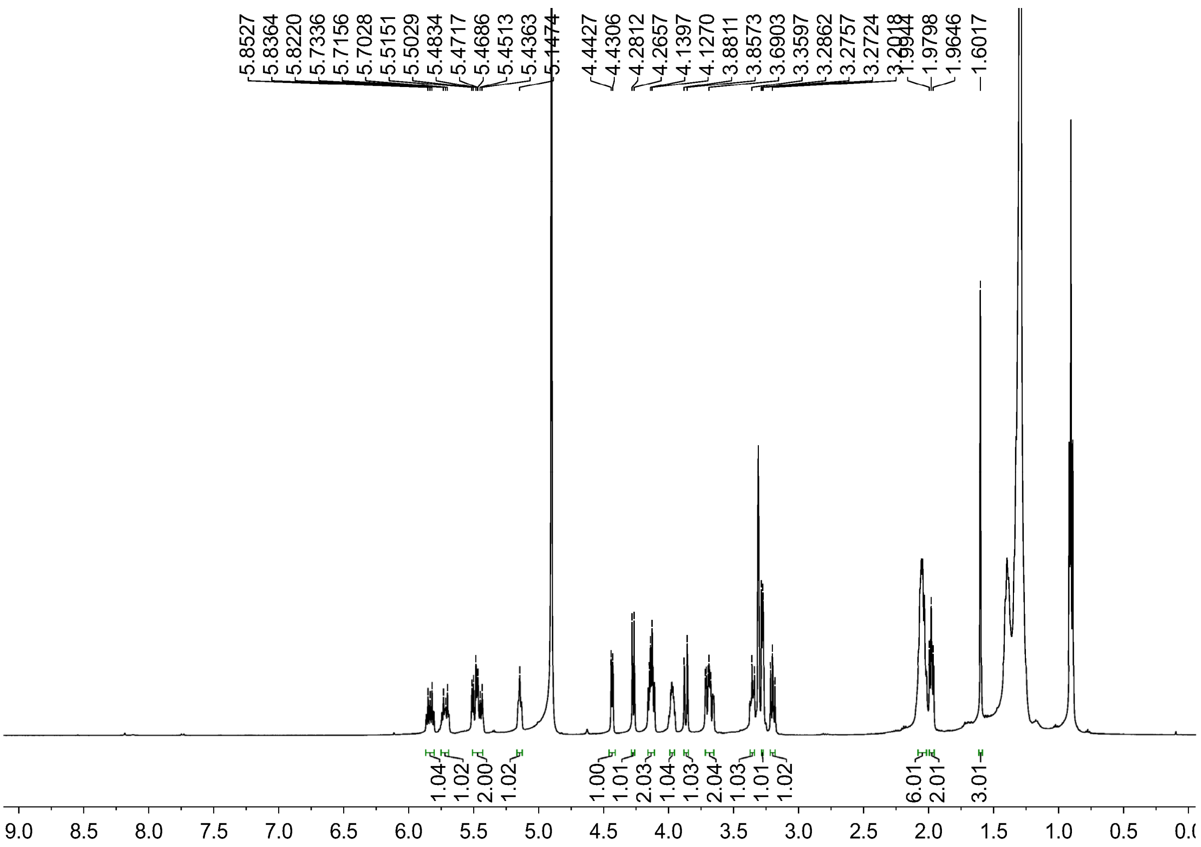

Figure S7. ^{1}H NMR (500 MHz, methanol-d_4) of compound **1**.

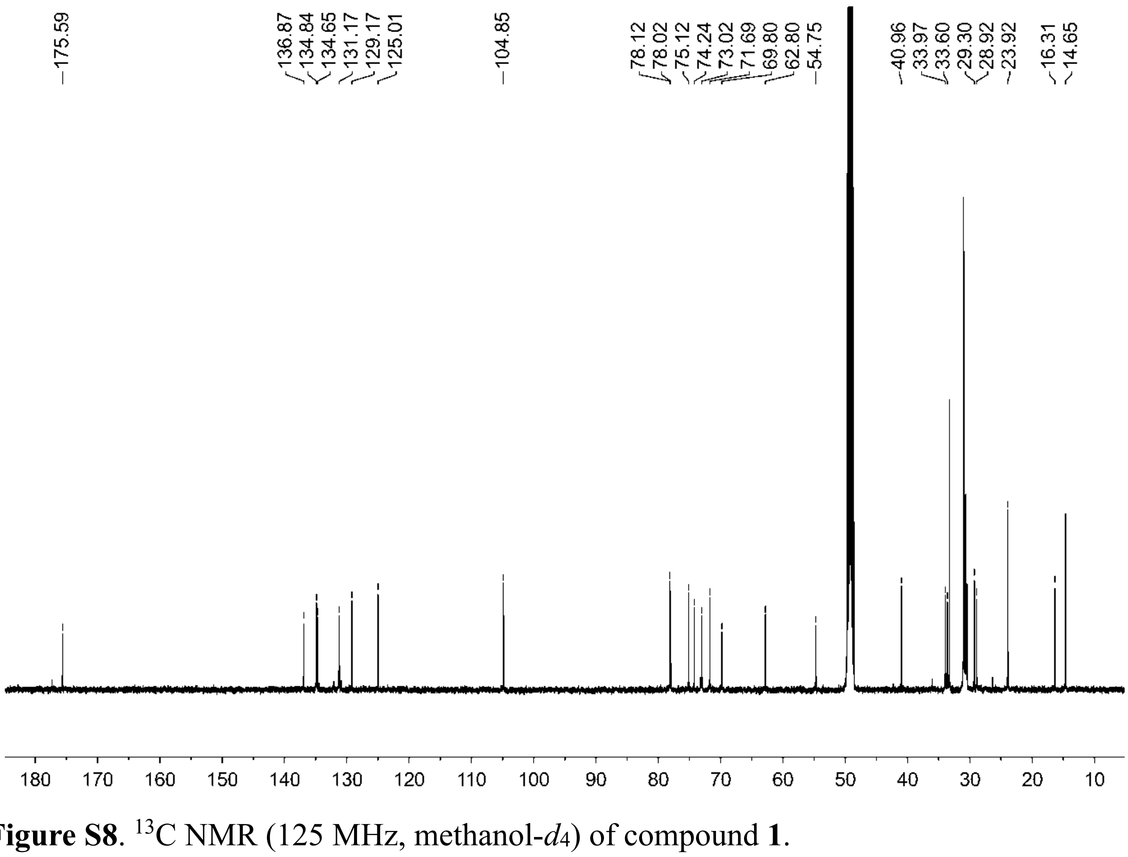

Figure S8. ^{13}C NMR (125 MHz, methanol-d_4) of compound **1**.

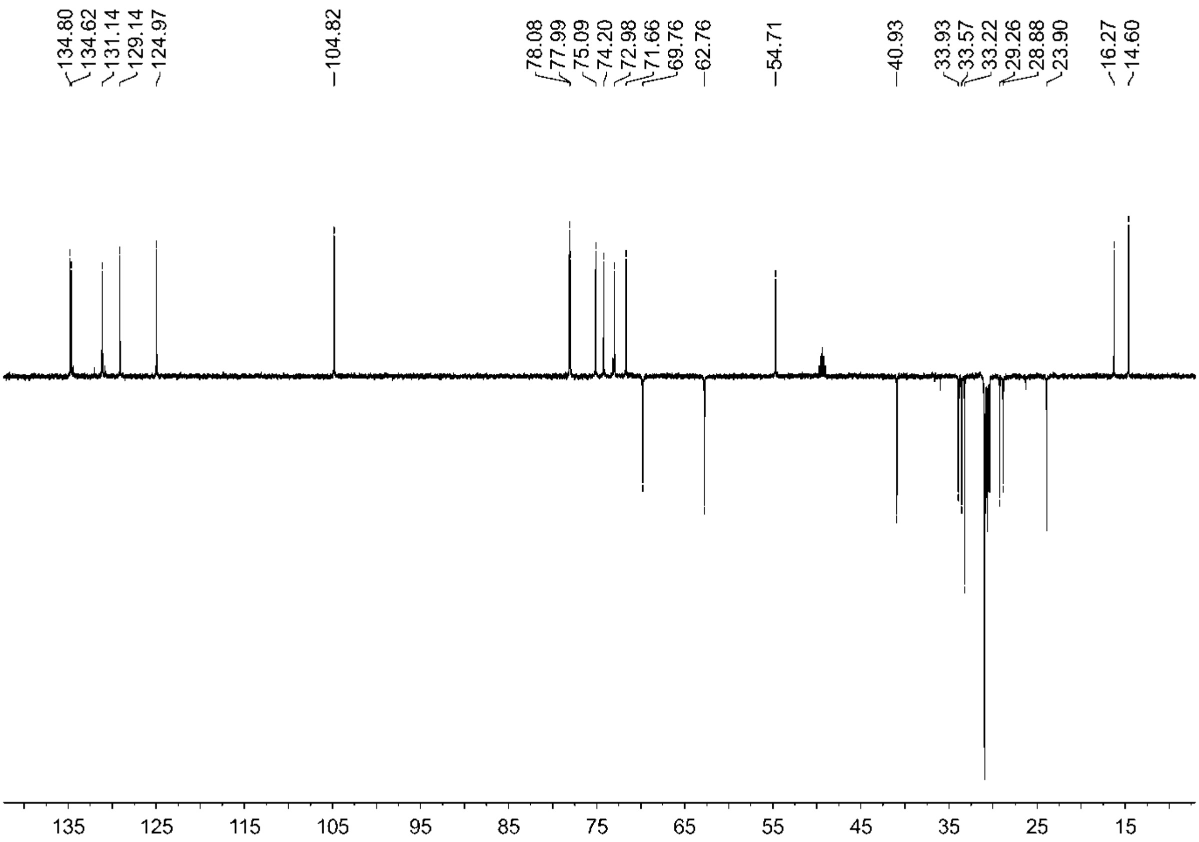

Figure S9. DEPT135 (125 MHz, methanol-d_4) of compound **1**.

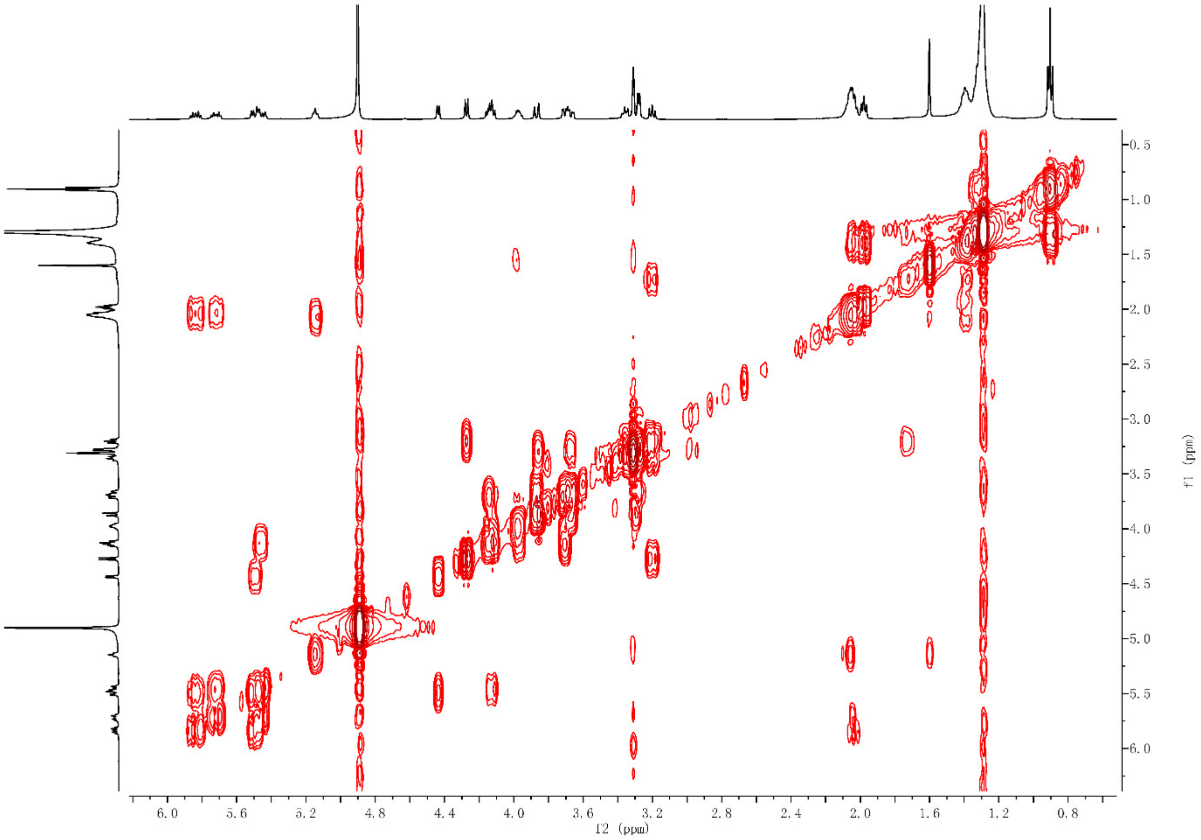

Figure S10. $^1H-^1H$ COSY (500 MHz, methanol-d_4) of compound **1**.

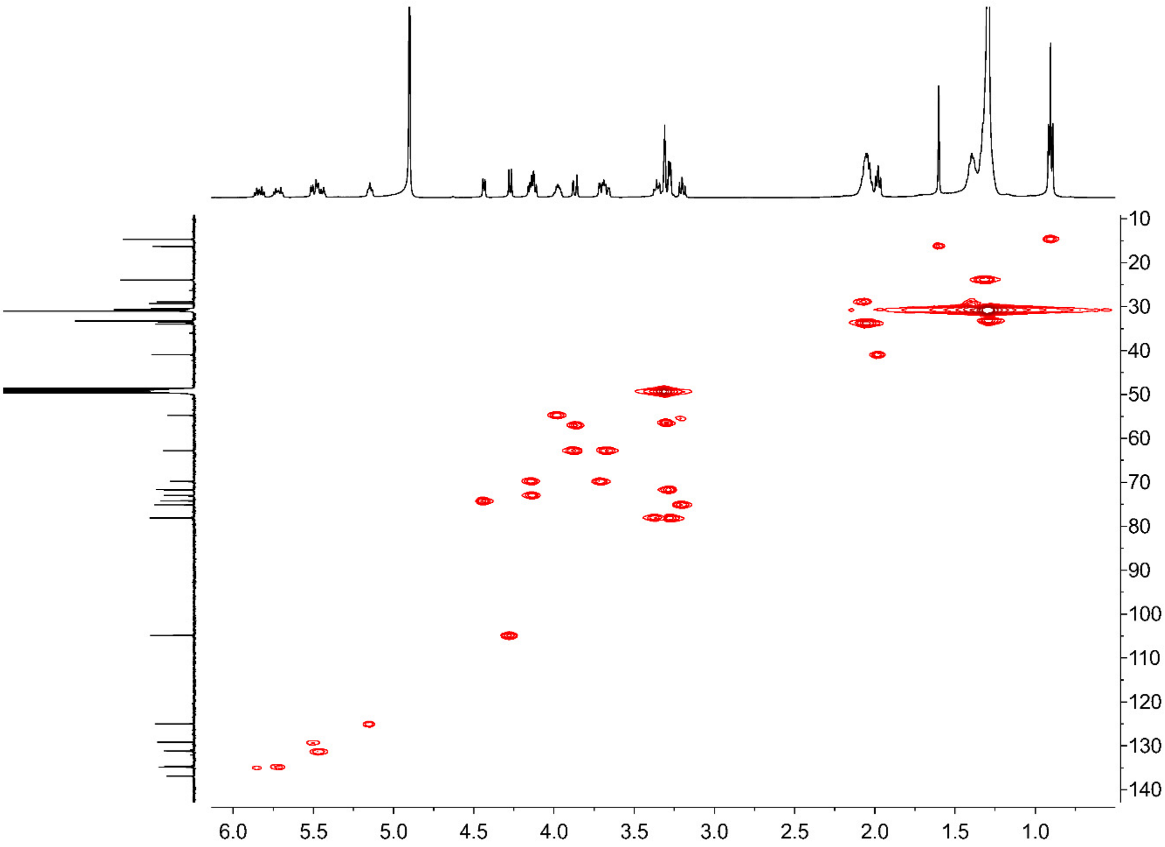

Figure S11. HSQC (methanol-*d*4) of compound **1** (^{1}H: 500 MHz, ^{13}C: 125 MHz).

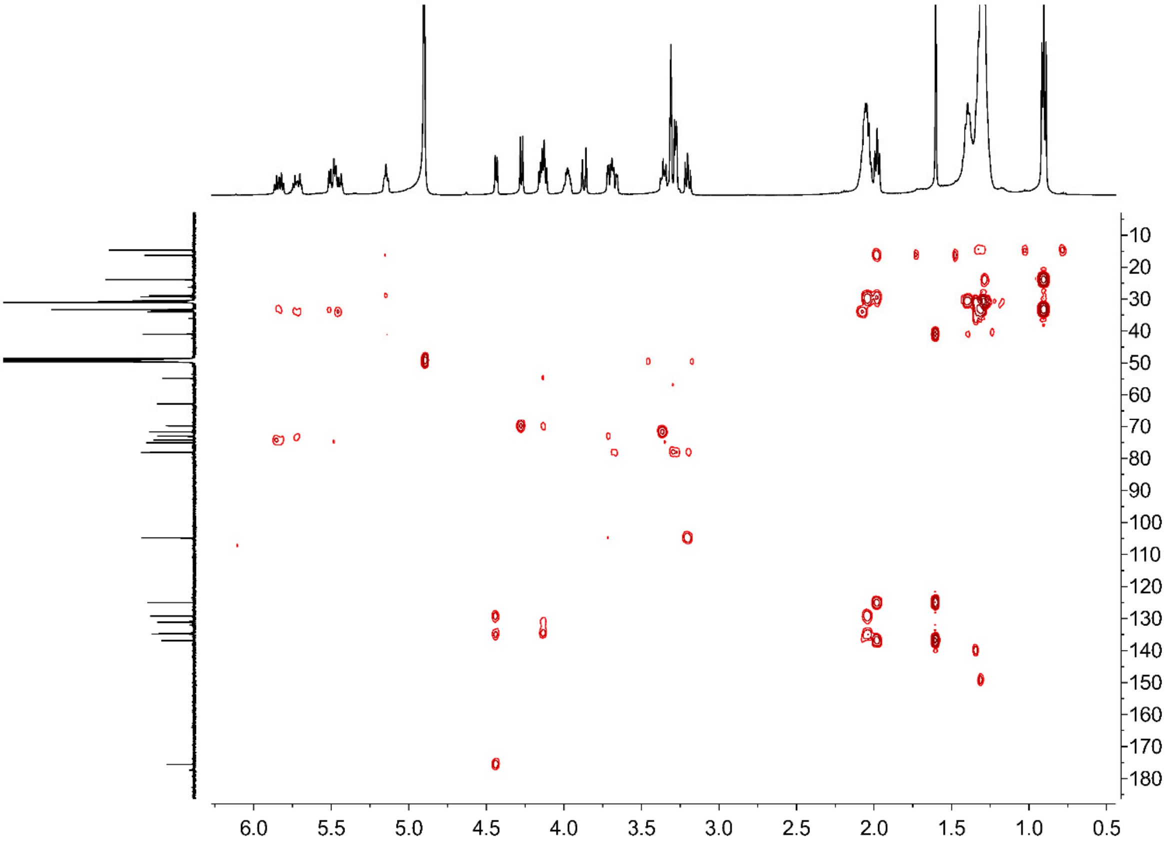

Figure S12. HMBC (methanol-*d*4) of compound **1** (^{1}H: 500 MHz, ^{13}C: 125 MHz).

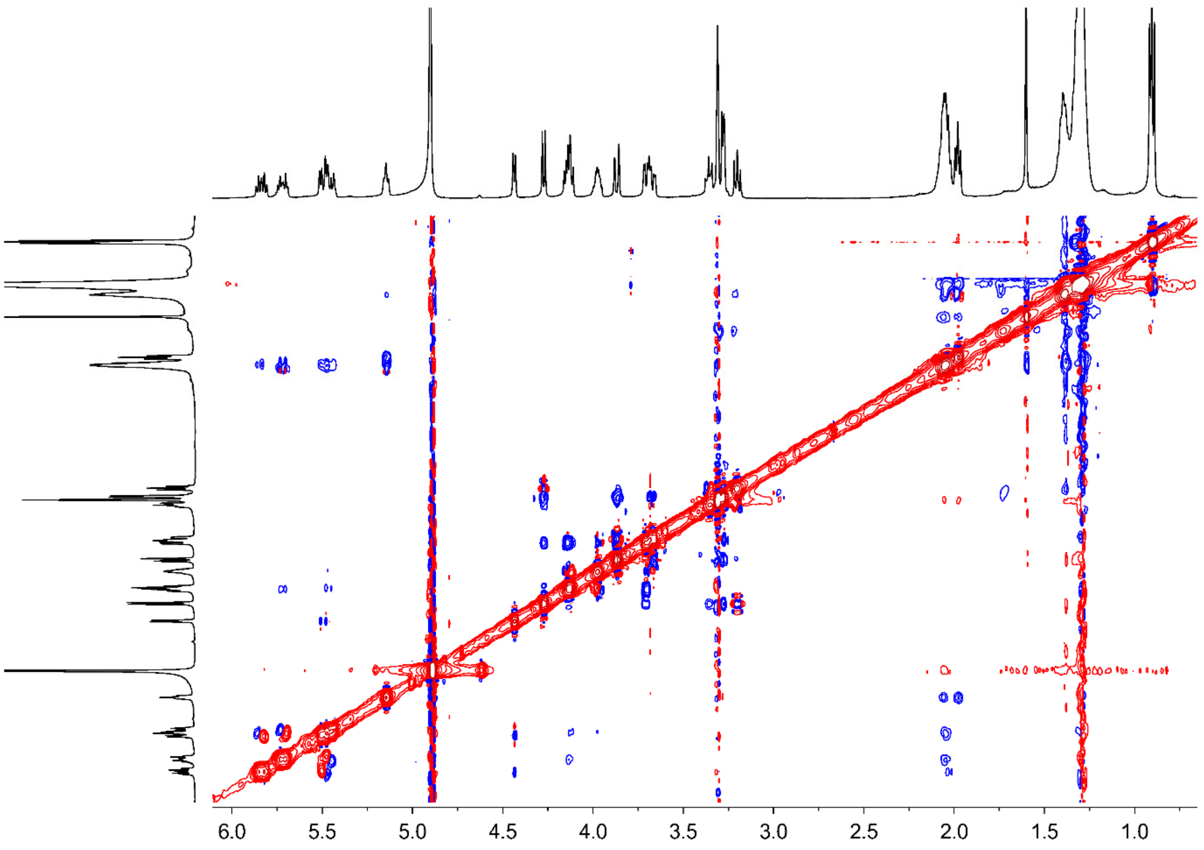

Figure S13. NOESY (500 MHz, methanol-d_4) of compound **1**.

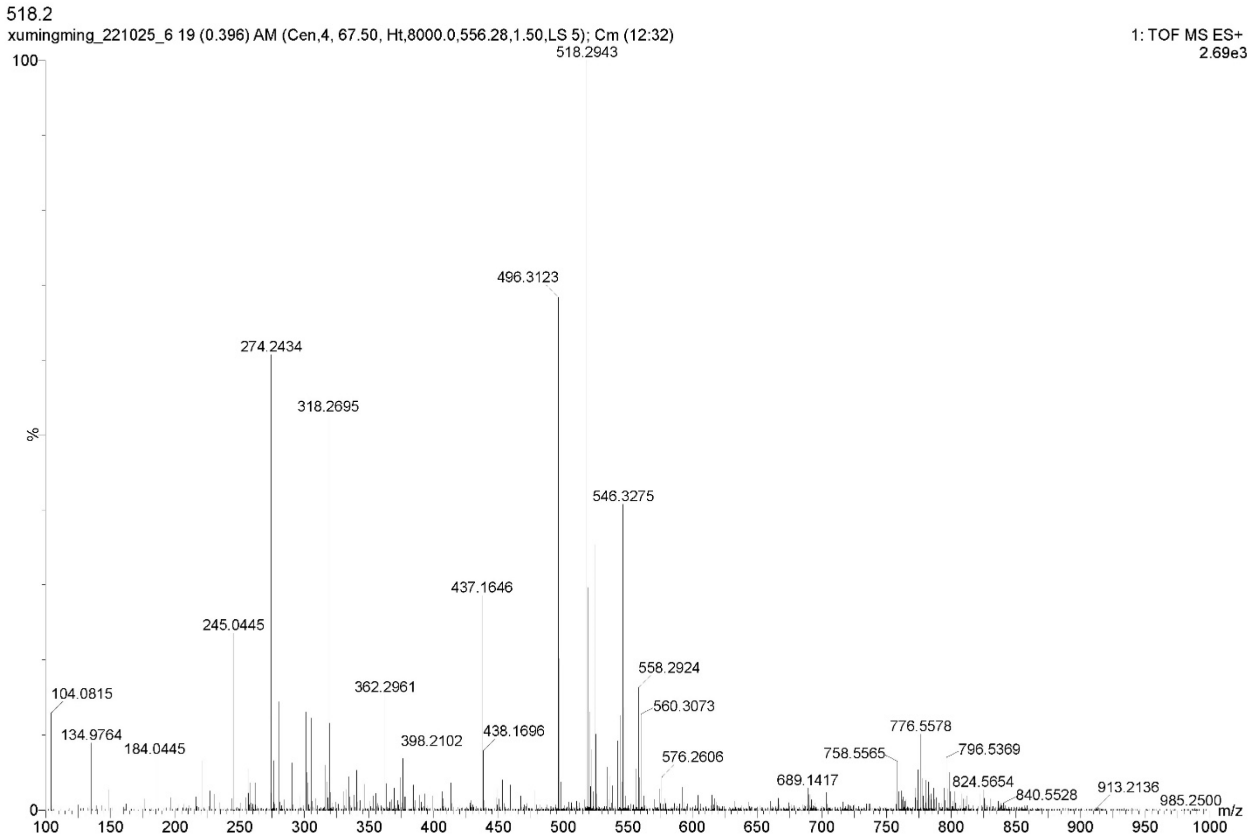

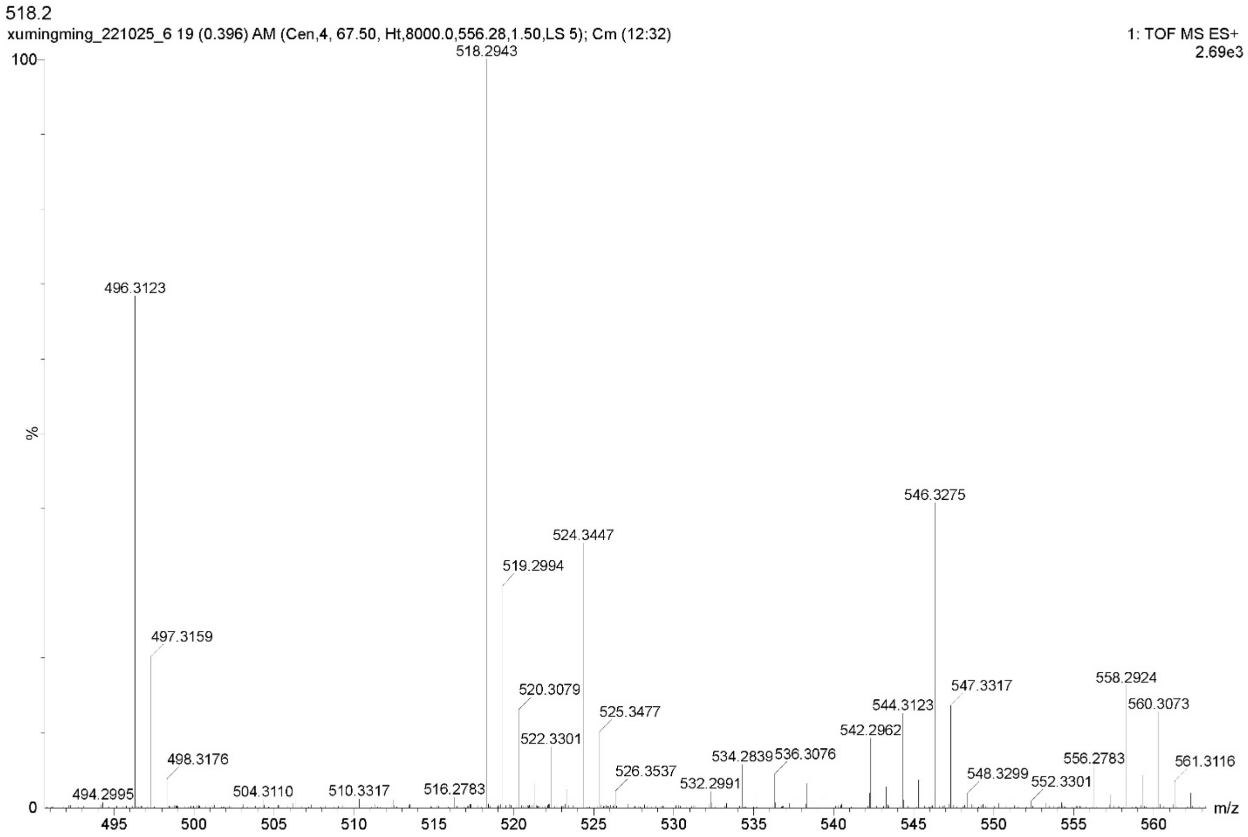

Figure S14. (+)-HR-ESI-MS of compound **1**.

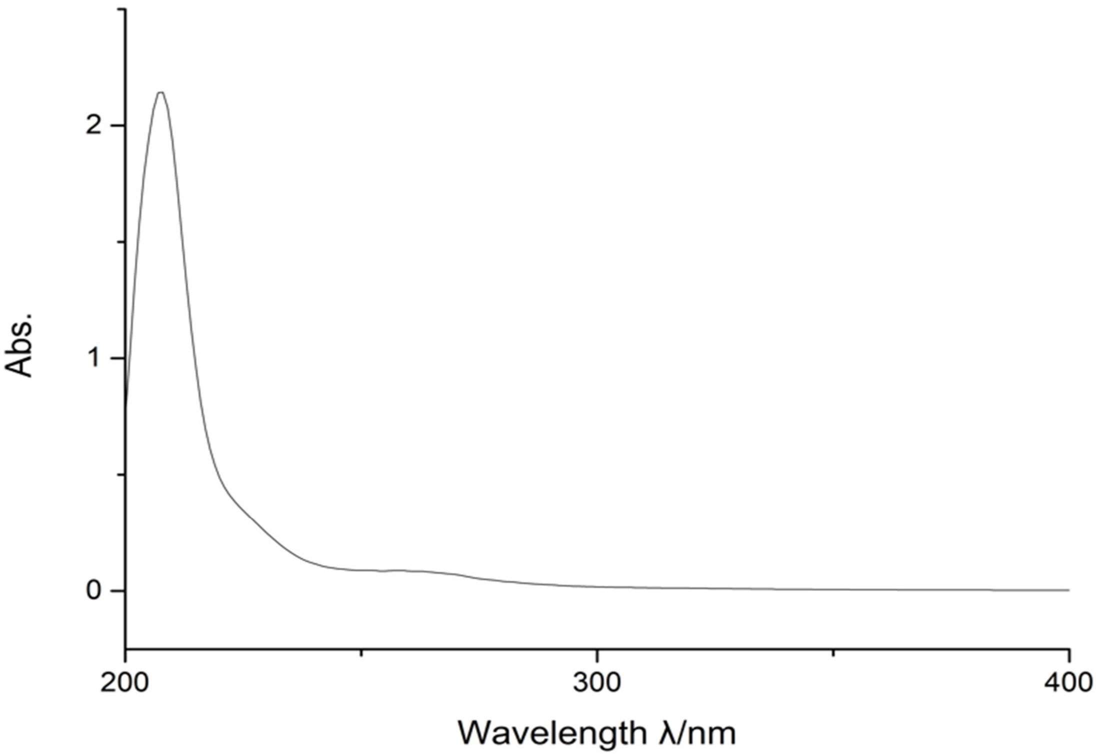

Figure S15. UV of compound **1**.

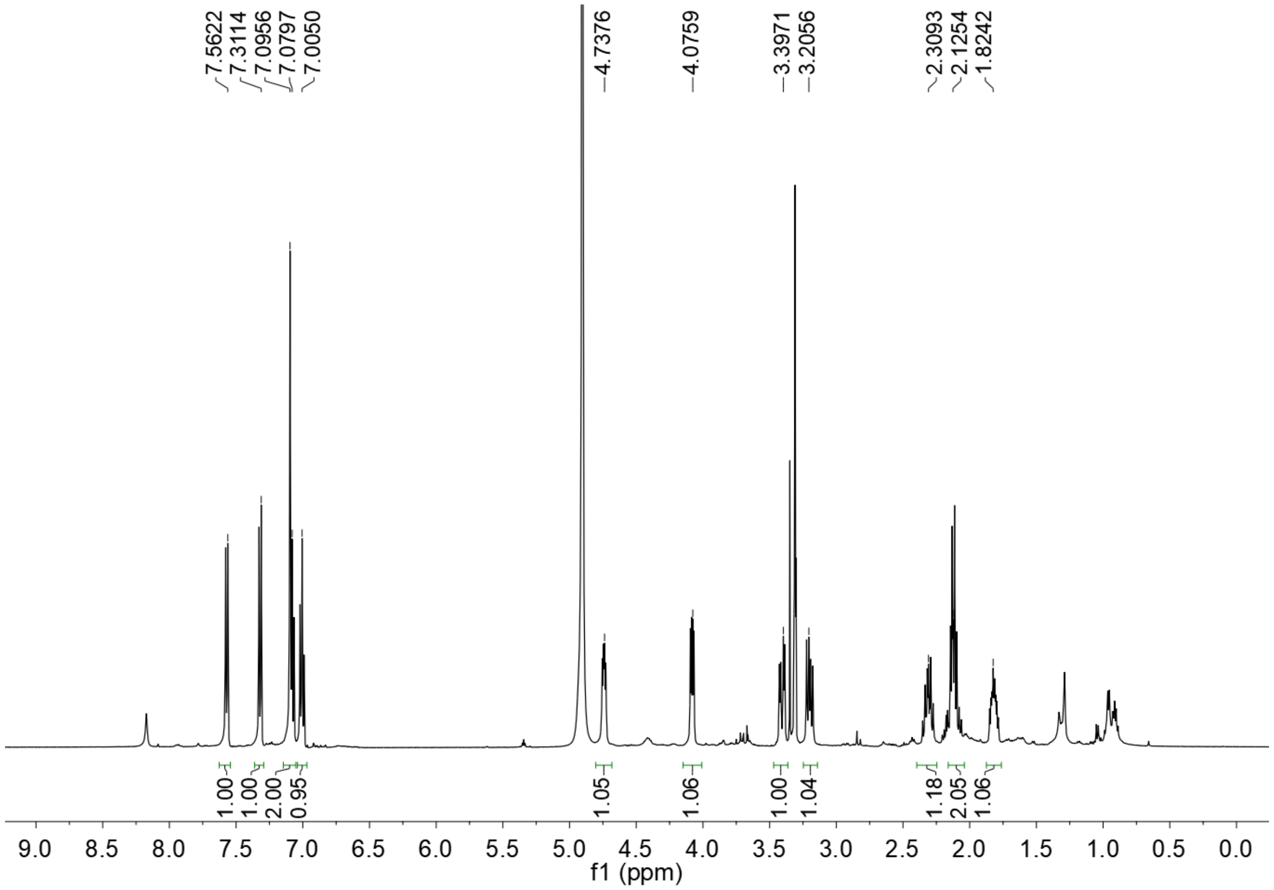

Figure S16. ^{1}H NMR (500 MHz, methanol-d_4) of compound **2**.

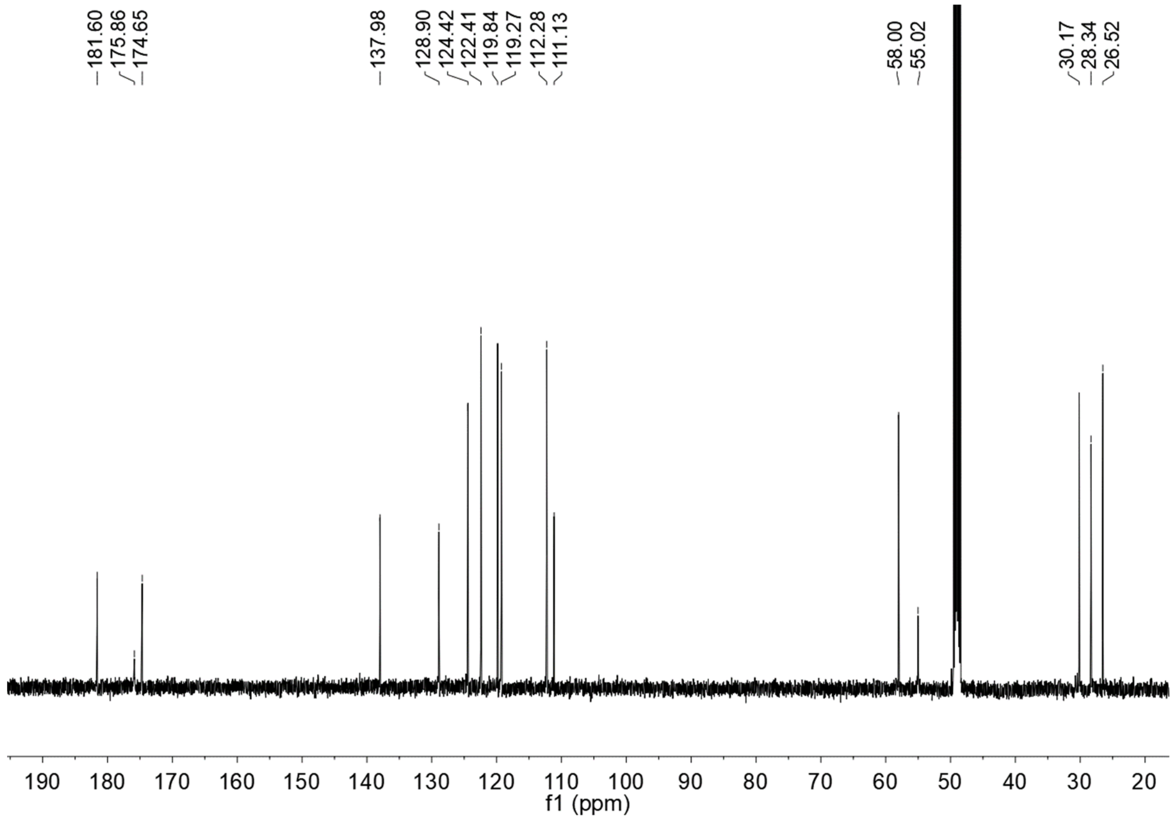

Figure S17. ^{13}C NMR (125 MHz, methanol-d_4) of compound **2**.

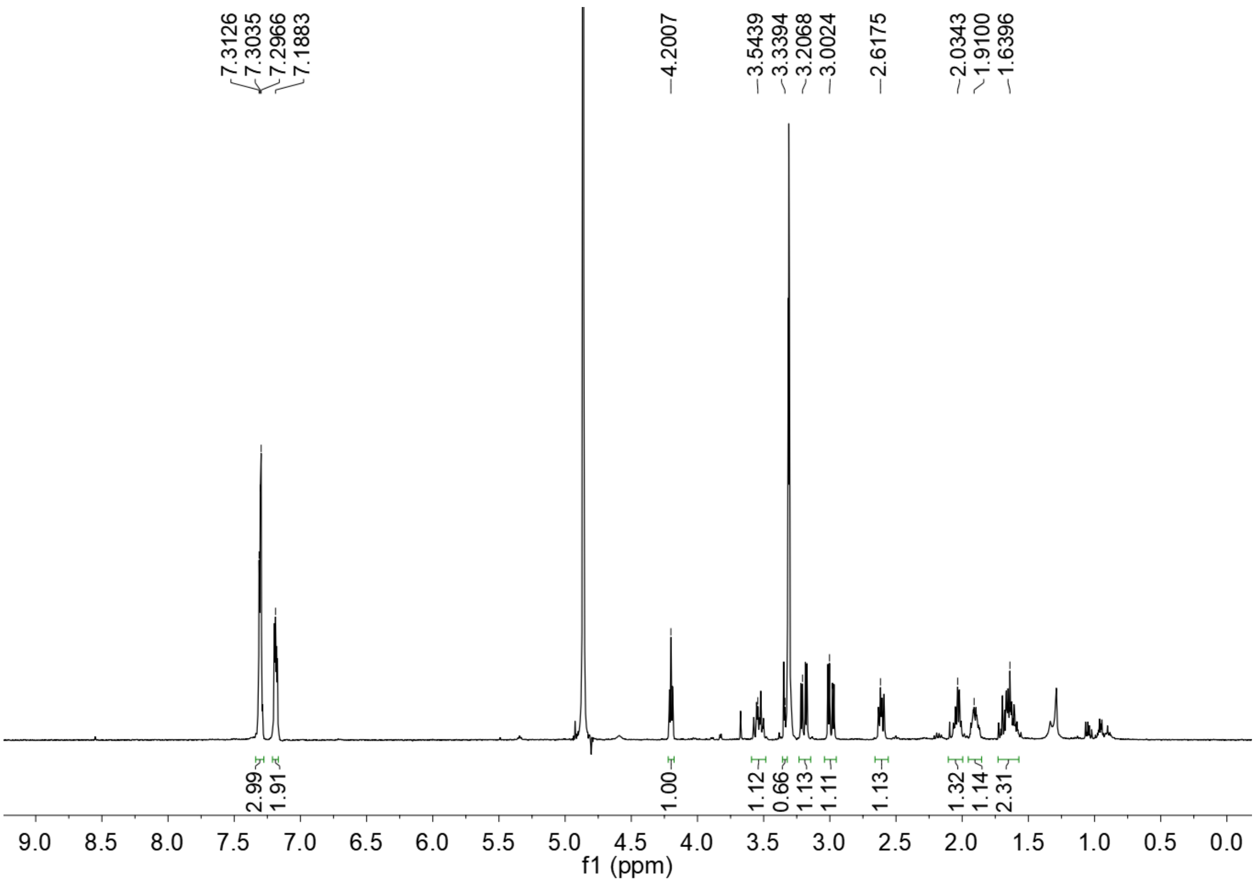

Figure S18. ^{1}H NMR (500 MHz, methanol-d_4) of compound **3**.

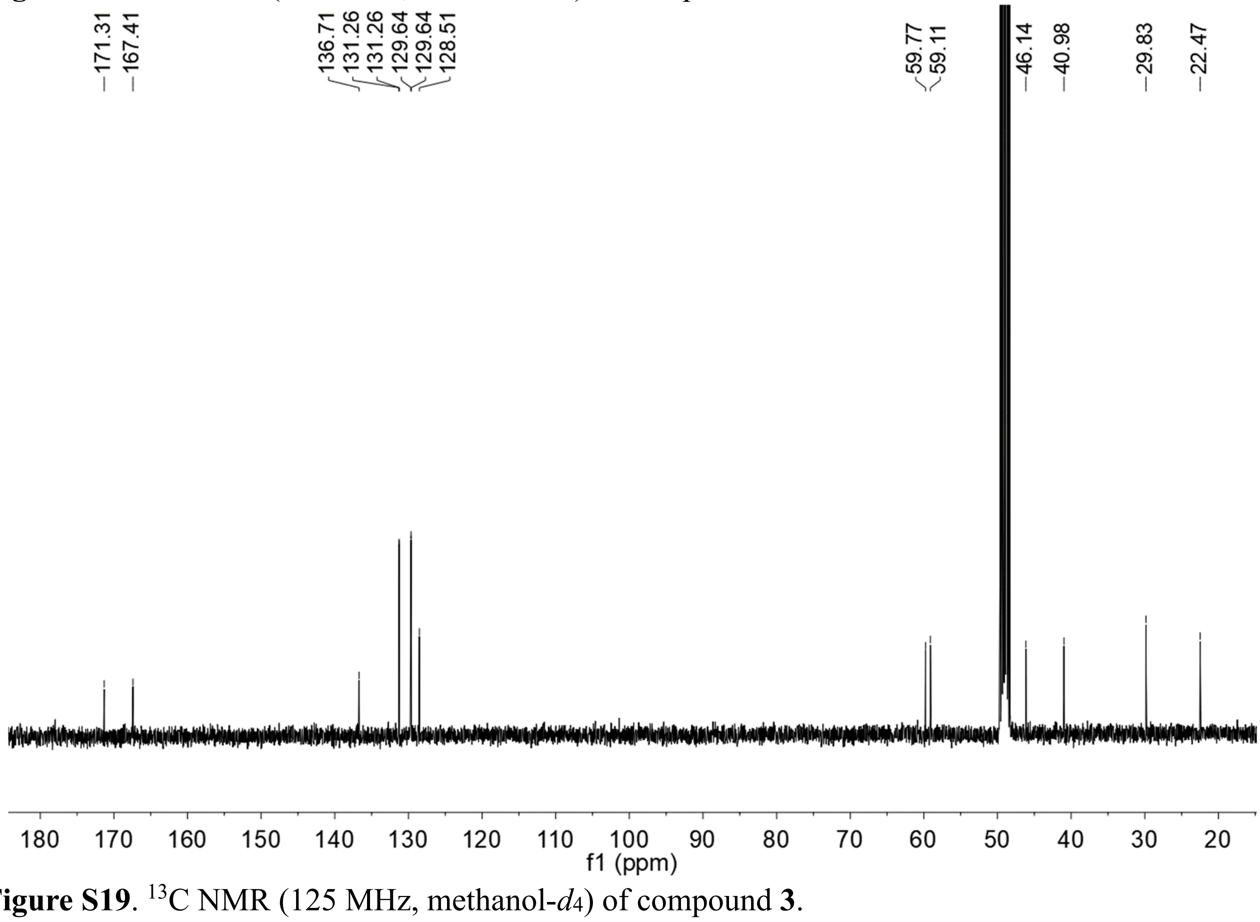

Figure S19. ^{13}C NMR (125 MHz, methanol-d_4) of compound **3**.

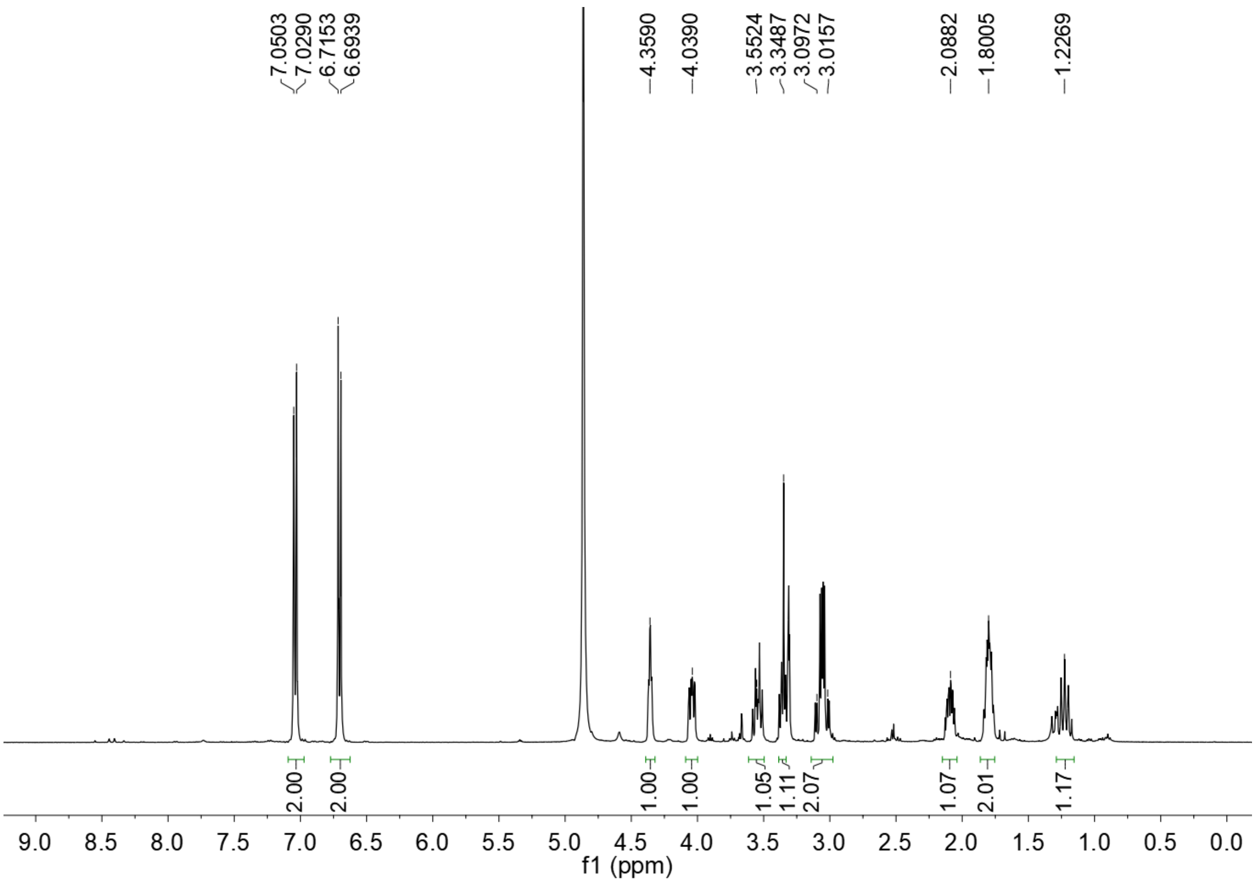

Figure S20. ^{1}H NMR (500 MHz, methanol-d_4) of compound **4**.

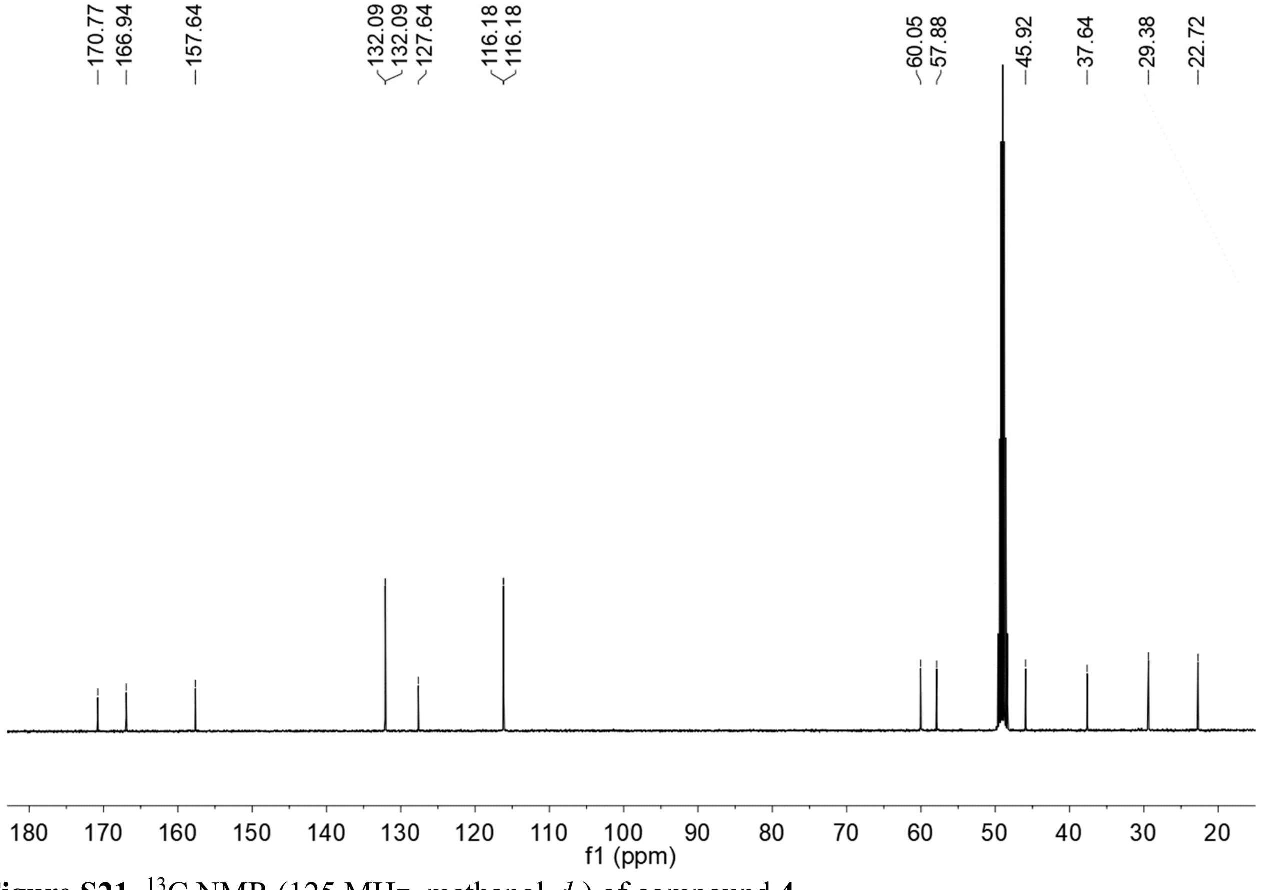

Figure S21. ^{13}C NMR (125 MHz, methanol-d_4) of compound **4**.

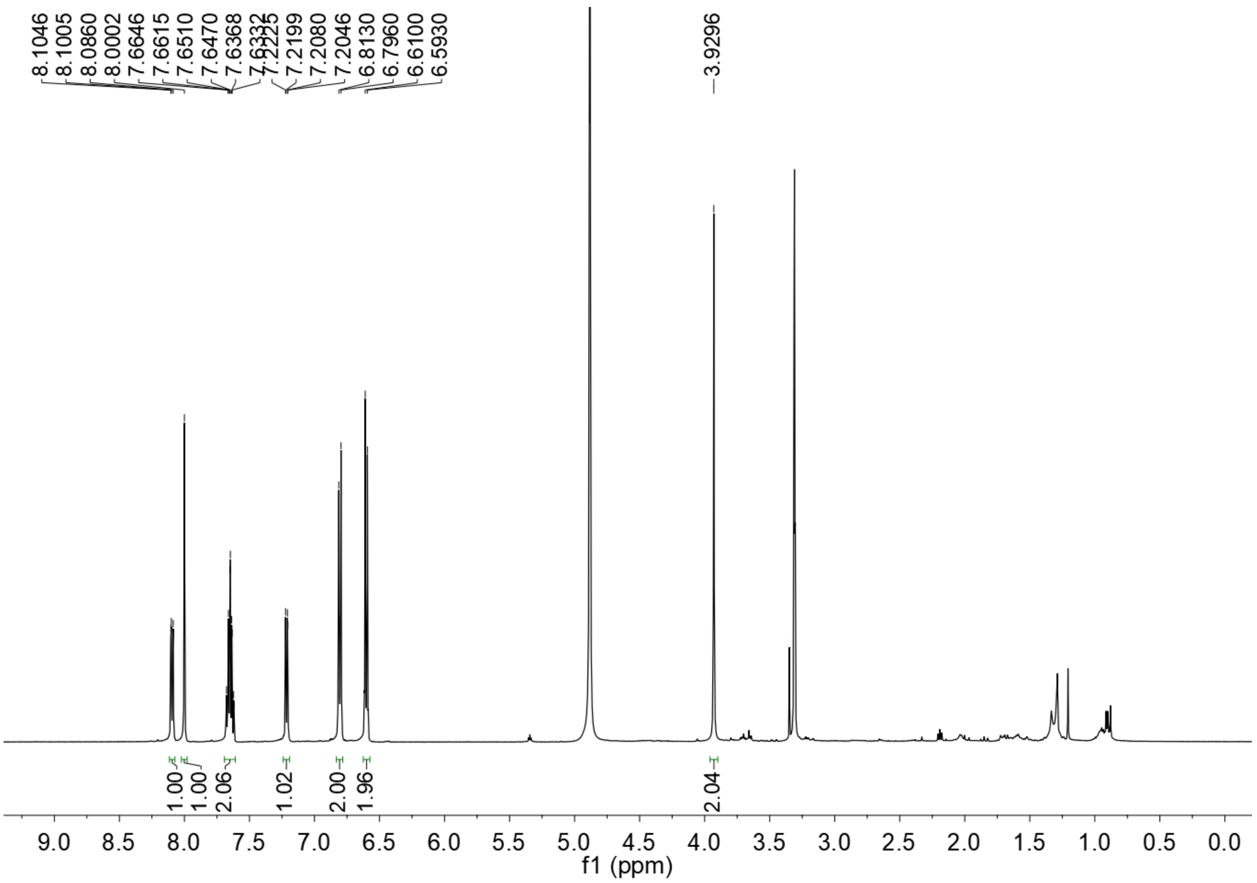

Figure S22. ^{1}H NMR (500 MHz, methanol-d_4) of compound **5**.

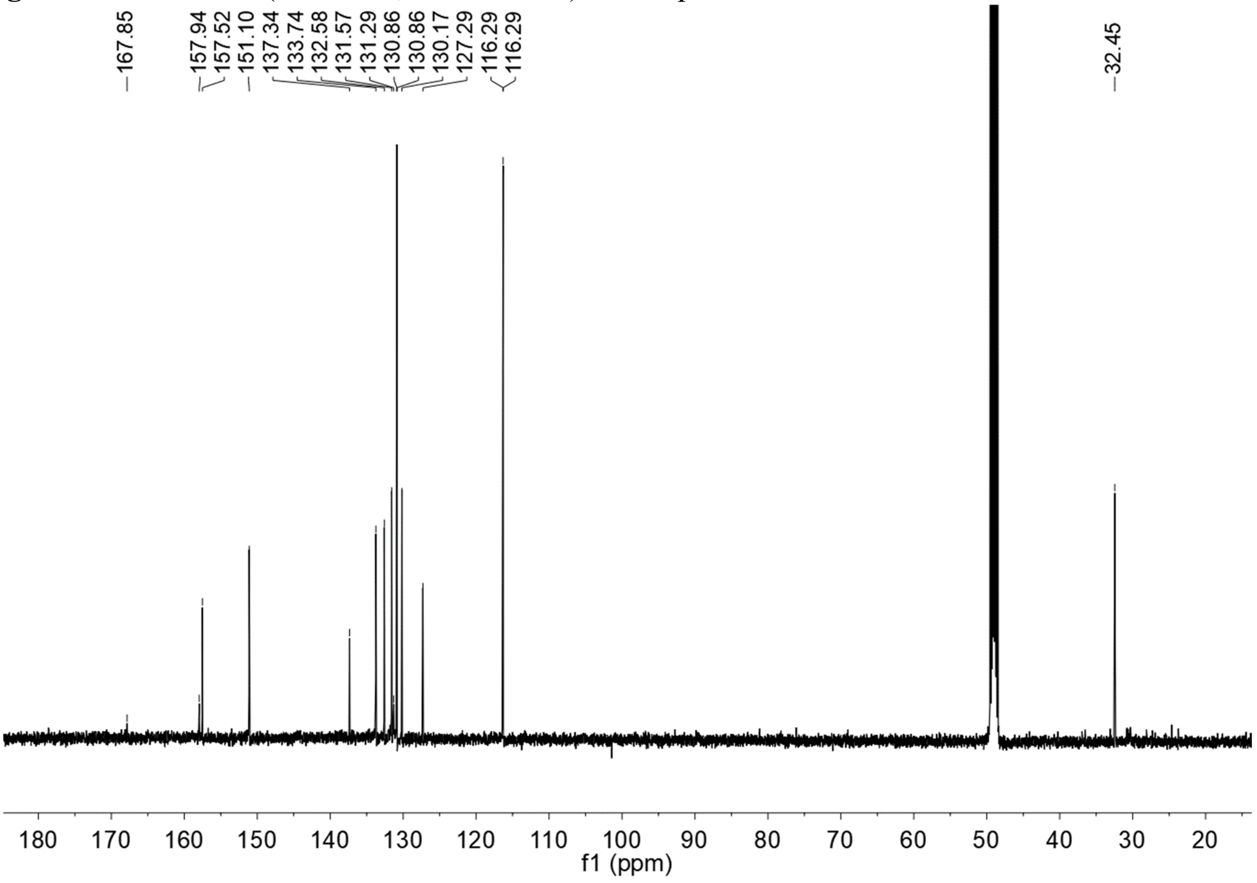

Figure S23. ^{13}C NMR (125 MHz, methanol-d_4) of compound **5**

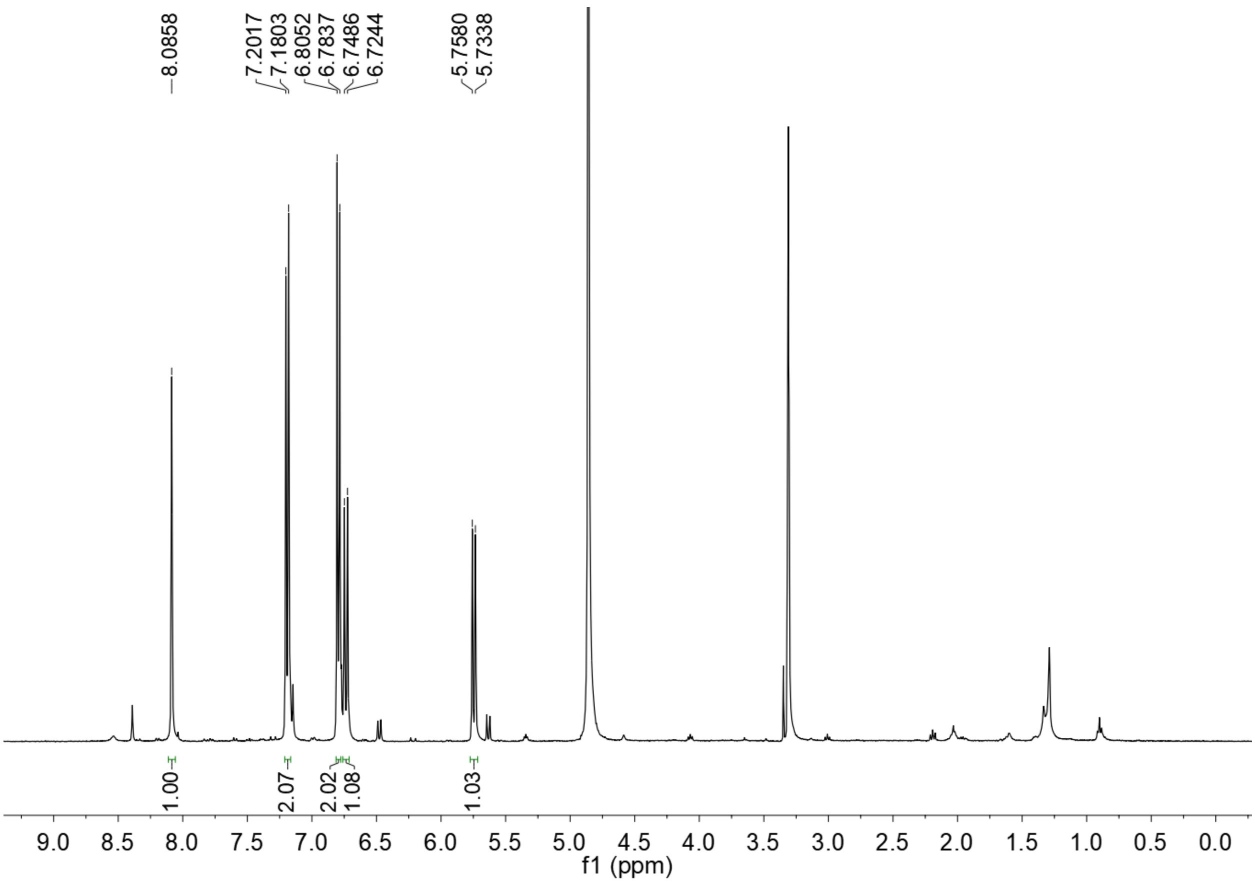

Figure S24. ^{1}H NMR (500 MHz, methanol-d_4) of compound **6**.

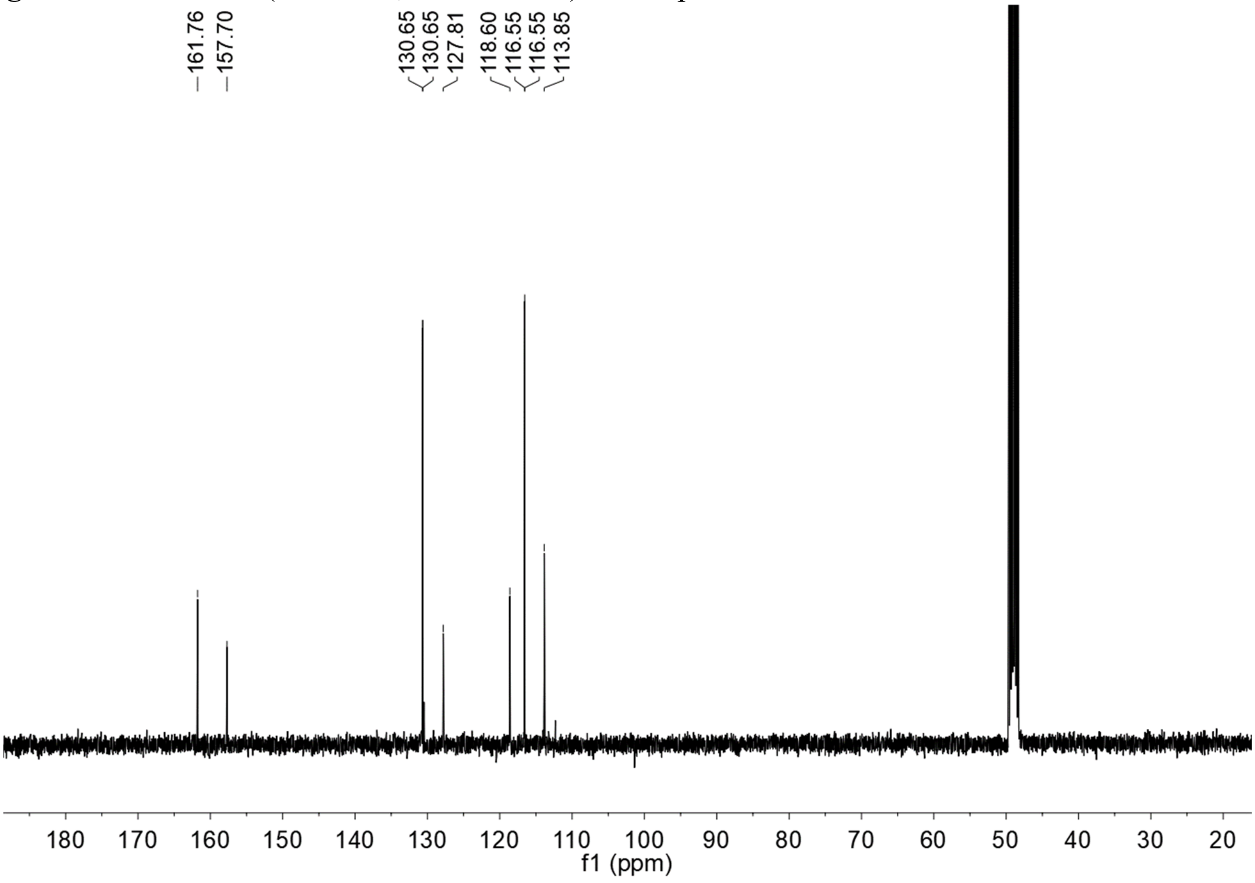

Figure S25. ^{13}C NMR (125 MHz, methanol-d_4) of compound **6**.

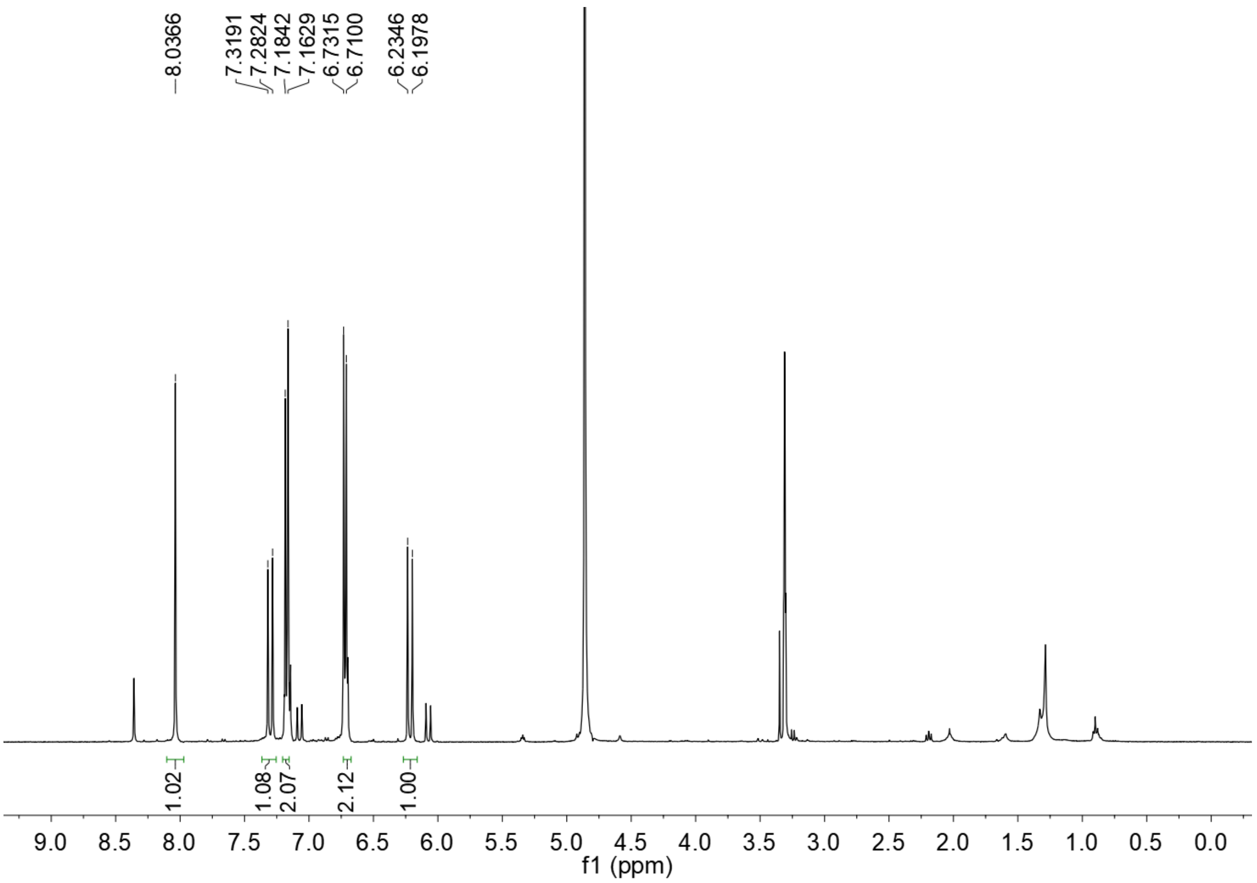

Figure S26. ^{1}H NMR (500 MHz, pyridine-d_5) of compound **7**.

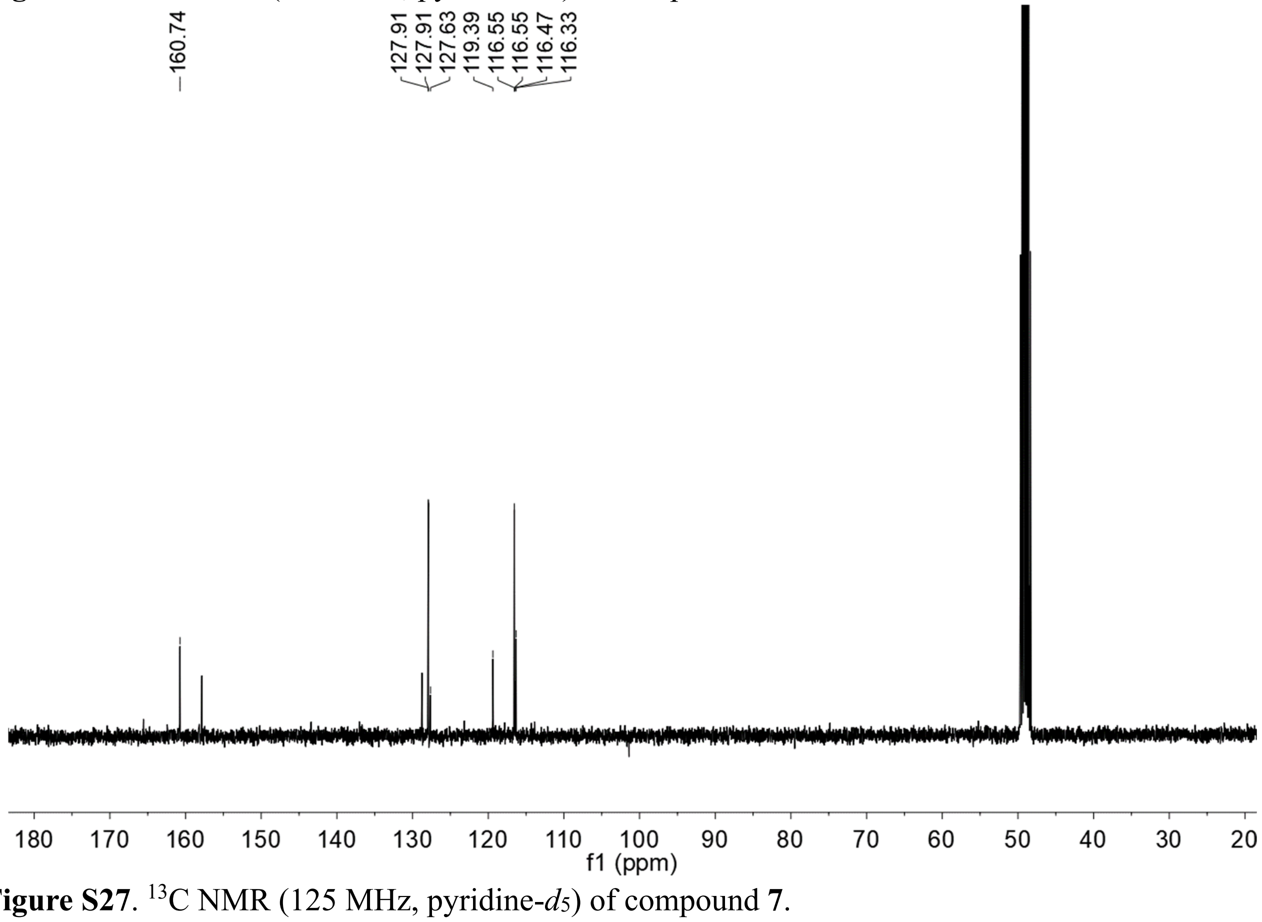

Figure S27. ^{13}C NMR (125 MHz, pyridine-d_5) of compound **7**.

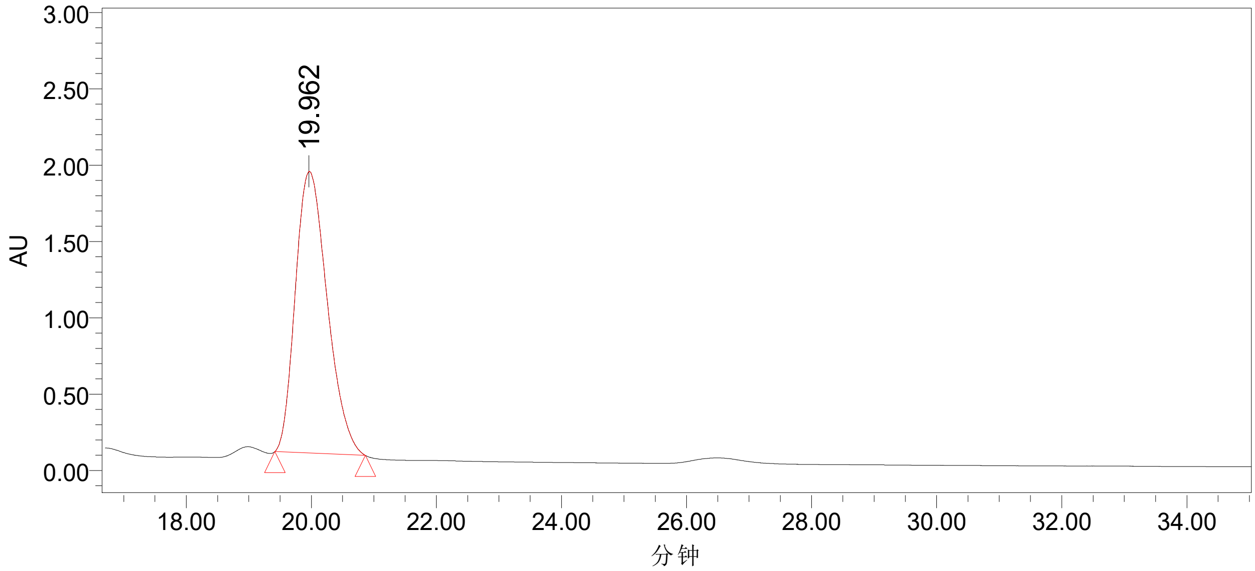

Figure S28. HPLC chromatogram of the derivative of the standard *L*-Glucose.

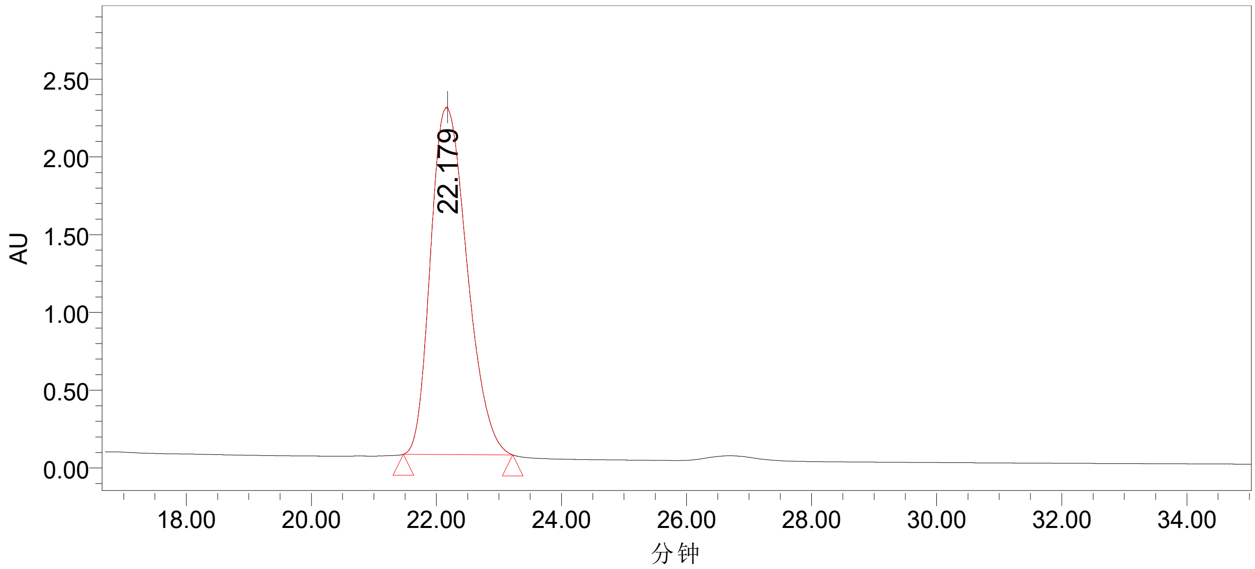

Figure S29. HPLC chromatogram of the derivative of the standard *D*-Glucose.

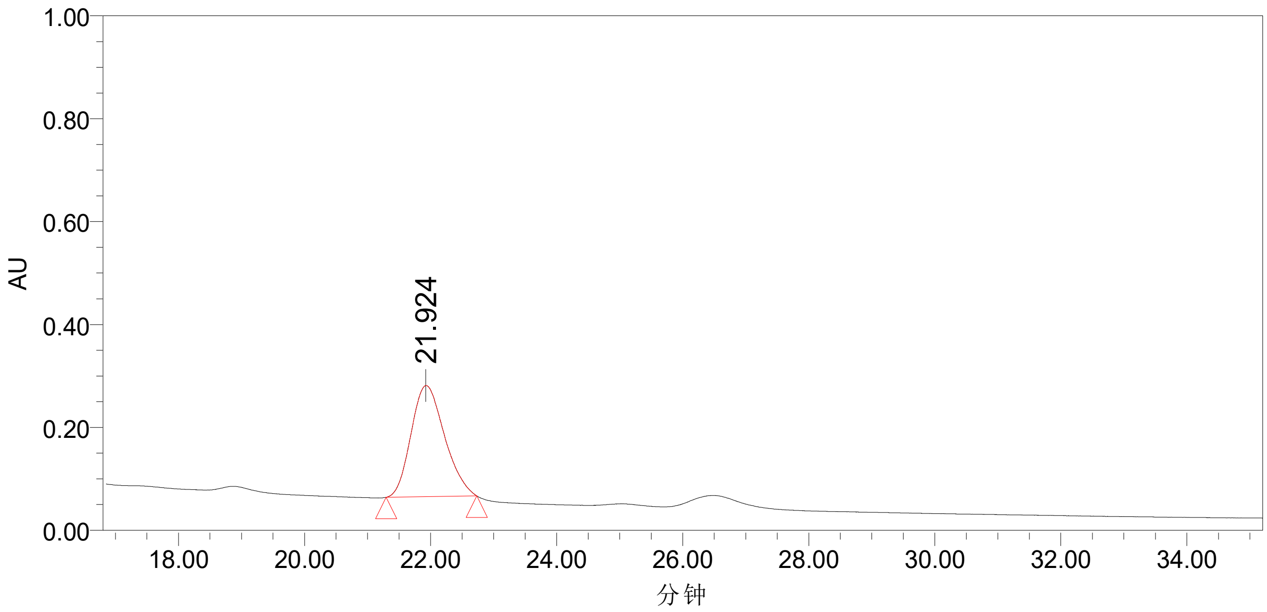

Figure S30. HPLC chromatogram of derivative of the hydrolysate of compound **1**.

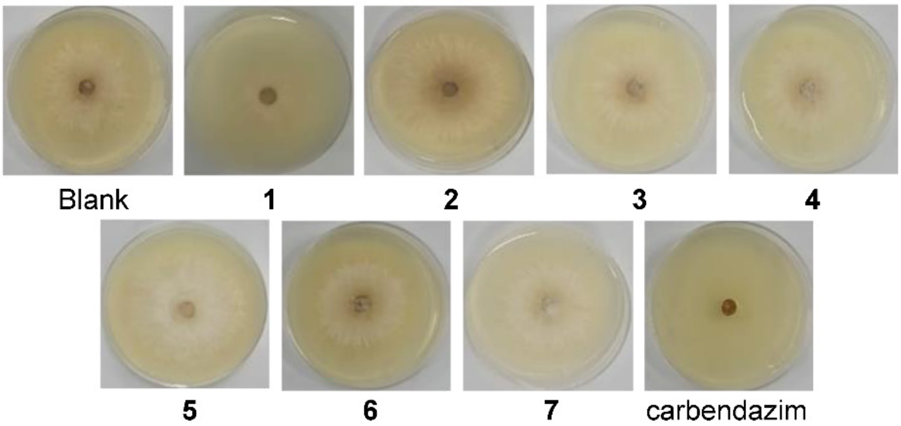

Figure S31. Compounds 1−7 and carbendazim (100 μM) inhibit the growth of *Rhizoctonia solani.*

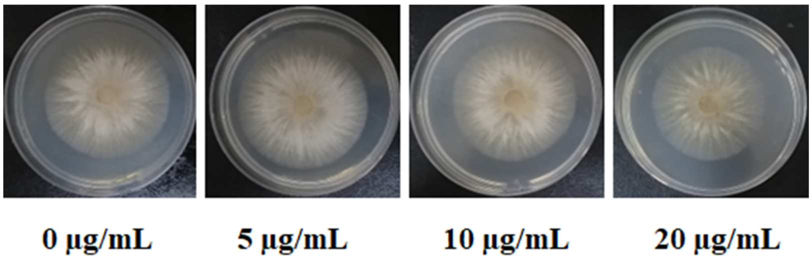

Figure S32. Growth inhibition of *Rhizoctonia solani* with different concentrations of compound **1**.
